# Fatigue-resistant adhesion of hydrogels

**DOI:** 10.1038/s41467-020-14871-3

**Published:** 2020-02-26

**Authors:** Ji Liu, Shaoting Lin, Xinyue Liu, Zhao Qin, Yueying Yang, Jianfeng Zang, Xuanhe Zhao

**Affiliations:** 1 Department of Mechanical and Energy Engineering, Southern University of Science and Technology, Shenzhen, 518055 China; 2 Department of Mechanical Engineering, Massachusetts Institute of Technology, Cambridge, MA 02139 USA; 3 Department of Civil and Environmental Engineering, Syracuse University, Syracuse, NY 13244 USA; 4 Syracuse Biomaterials Institute, Syracuse University, Syracuse, NY 13244 USA; 5 School of Optical and Electronic Information and Wuhan National Laboratory for Optoelectronics, Huazhong University of Science and Technology, Wuhan, 430074 China; 6 Department of Civil and Environmental Engineering, Massachusetts Institute of Technology, Cambridge, MA 02139 USA

**Keywords:** Biomedical engineering, Bioinspired materials, Biomedical materials

## Abstract

The adhesion of soft connective tissues (tendons, ligaments, and cartilages) on bones in many animals can maintain high toughness (∽800 J m^−2^) over millions of cycles of mechanical loads. Such fatigue-resistant adhesion has not been achieved between synthetic hydrogels and engineering materials, but is highly desirable for diverse applications such as artificial cartilages and tendons, robust antifouling coatings, and hydrogel robots. Inspired by the nanostructured interfaces between tendons/ligaments/cartilages and bones, we report that bonding ordered nanocrystalline domains of synthetic hydrogels on engineering materials can give a fatigue-resistant adhesion with an interfacial fatigue threshold of 800 J m^−2^, because the fatigue-crack propagation at the interface requires a higher energy to fracture the ordered nanostructures than amorphous polymer chains. Our method enables fatigue-resistant hydrogel coatings on diverse engineering materials with complex geometries. We further demonstrate that the fatigue-resistant hydrogel coatings exhibit low friction and low wear against natural cartilages.

## Abstract (web summary)

Fatigue-resistant adhesion is of interest for a range of applications, but has been limited in synthetic hydrogels. Here, the authors report on a synthetic hydrogel with ordered nanocrystalline domains resulting in high fatigue-resistant adhesion and demonstrate the coating of different surfaces.

## Introduction

Tough adhesions between hydrogels and engineering materials have been achieved by covalently anchoring polymer chains of tough hydrogels on solid surfaces (Supplementary Fig. 1a)^1^. When the hydrogel is peeled from the solid under a single cycle of the mechanical load, the energy required for fracturing anchored polymer chains and the energy dissipated in deforming the bulk hydrogel synergistically give an interfacial toughness over 1000 J m^−2^. However, such tough hydrogel adhesion suffers from fatigue failure over multiple cycles of mechanical loads, in which the effect of bulk dissipation has been depleted (Supplementary Fig. 1b). The resultant interfacial fatigue threshold (i.e., the minimal fracture energy at which interfacial crack propagation occurs under cyclic loads) is equal to the energy for fracturing a single layer of bonded amorphous polymer chains, on the order of 1–100 J m^−2^ ^2–7^.

**Fig. 1:**
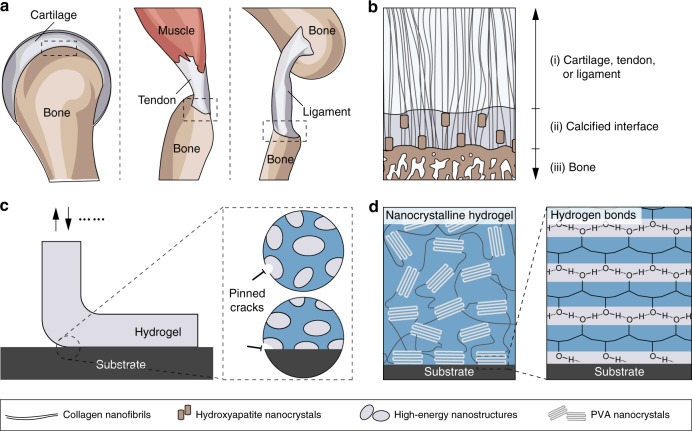
Bioinspired design of fatigue-resistant hydrogel adhesion. **a** Schematic illustration of the fatigue-resistant adhesions between soft connective tissues (cartilage, tendon, and ligament) and bones. **b** The transitional interface from uncalcified collagen nanofibrils (i) to calcified nanofibers (ii) to the bone (iii). The nanostructured composites of aligned collagen nanofibrils and ordered hydroxyapatite nanocrystals contribute to the fatigue-resistant adhesions of the cartilages, tendons, and ligaments to the bones. **c** Bioinspired fatigue-resistant adhesion of synthetic hydrogels by anchoring ordered high-energy nanostructures (e.g., nanocrystalline domains) on engineering materials. These high-energy nanostructures (e.g., nanocrystalline domains) can effectively pin the crack propagation both at the interface and within the bulk hydrogels, since they require a much higher energy for fatigue-crack propagation than the corresponding amorphous polymer chains. **d** Fatigue-resistant adhesion of poly(vinyl alcohol) (PVA) hydrogel to substrates through the anchorage of ordered nanocrystalline domains on the substrates with hydrogen bonds.

In nature, the adhesions of tendons, ligaments, and cartilages to bones are commonly achieved through a transitional interface (Fig. 1a), from the uncalcified collagen nanofibrils (i) to the calcified collagen nanofibrils (ii) to the bones (iii) (Fig. 1b)^8^. At the interface (ii), nanostructured composites of aligned collagen nanofibrils and ordered hydroxyapatite nanocrystals^8–13^ are anchored on the bones, leading to fatigue-resistant adhesions of tendons, ligaments, and cartilages to the bones (Fig. 1b)^12^. For example, the cartilage–bone interface in the human knee joint can sustain compressive stresses of 1 MPa along with an interfacial toughness around 800 J m^−2^ over 1 million cycles of loading per year^14–16^.

Here, we propose a bioinspired strategy to achieve fatigue-resistant adhesions of synthetic hydrogels by anchoring ordered nanostructures (e.g., nanocrystalline domains) on engineering materials, since the ordered nanostructures require a much higher energy for fatigue-crack propagation than the corresponding amorphous polymer chains (Fig. 1c). To test this strategy, we choose poly(vinyl alcohol) (PVA) hydrogels as a model material system, which can readily form nanostructures (e.g., nanocrystalline domains and nanofibrils) with tuneable crystallinity. The anchorage of nanocrystalline domains on solid substrates through dry-annealing treatment gives a remarkable fatigue-resistant adhesion between hydrogels and substrates, with an interfacial fatigue threshold of 800 J m^−2^. The fatigue-resistant hydrogel adhesion can potentially enable a number of applications such as robust hydrogel coatings on devices made of various materials and with various sizes and shapes. In particular, we demonstrate that the fatigue-resistant hydrogel coatings exhibit low friction and low wear against natural cartilages.

## Results

### Mechanical characterization of fatigue-resistant hydrogel adhesion

We first form the PVA hydrogels with low crystallinity on diverse solid substrates including glass, ceramics, titanium, aluminum, stainless steel, polyurethane (PU), and polydimethylsiloxane (PDMS) through a freeze–thawing process^7^. Then, we dry and anneal the samples to substantially increase the crystallinity within the hydrogels (see Methods for details)^7, 17^. The dry-annealing process also induces the formation of hydrogen bonds between the ordered nanocrystalline domains and the solid surfaces (Fig. 1d)^18^. As control samples, we fabricate tough hydrogel adhesion (e.g., polyacrylamide (PAAm)–alginate hydrogel) and common hydrogel adhesion (e.g., PAAm hydrogel and polyacrylic acid (PAA) hydrogel) to solid substrates by covalently anchoring the polymer networks of hydrogels on the substrates (see Methods for details)^1^.

**Fig. 2:**
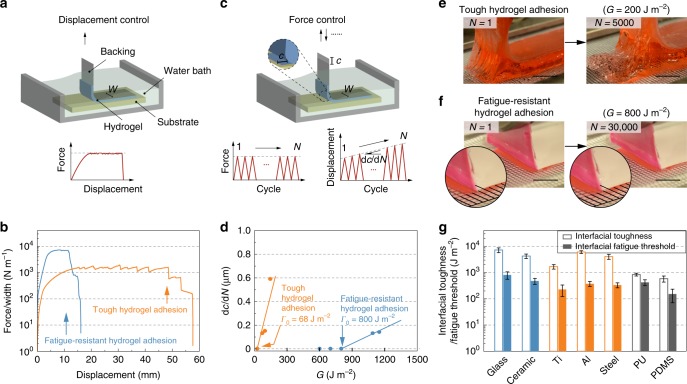
Mechanical characterization of fatigue-resistant hydrogel adhesion. **a** Schematic illustration of measuring nominal peeling force *F* versus displacement curve during a single cycle of loading within a water bath. **b** Representative curves of the peeling force per width of the hydrogel (*F*/*W*) versus displacement for the tough hydrogel adhesion (i.e., PAAm-alginate) and fatigue-resistant hydrogel adhesion (i.e., PVA) on a glass substrate. **c** Schematic illustration of measuring the interfacial crack extension *c* versus cycle number *N* during a cyclic peeling test at a peeling force of *F*_*a*_. **d** Plot of crack extension rate (d*c*/d*N*) versus applied energy release rate *G* = *F*_*a*_/*W* for the tough hydrogel adhesion and fatigue-resistant hydrogel adhesion on a glass substrate. The linear extrapolation to the *G*-axis gives the fatigue threshold *Γ*_0_. **e** Images of interfacial crack propagation during a cyclic peeling test for tough hydrogel adhesion with a thickness around 3 mm (swollen state) at an energy release rate of 200 J m^−2^, showing substantial interfacial crack propagation within 5000 cycles. **f** Images of interfacial crack propagation during a cyclic peeling test for fatigue-resistant hydrogel adhesion with a thickness around 100 μm (swollen state) at an energy release rate of 800 J m^−2^, showing no interfacial crack propagation within 30,000 cycles. **g** Summary of measured interfacial toughness and interfacial fatigue threshold for fatigue-resistant hydrogel adhesion on various substrates, including glass, ceramic, titanium (Ti), aluminum (Al), stainless steel, PU, and PDMS. Error bars = standard deviation (*n* = 3). Scale bars: 10 mm in (**e**, **f**).

Next, we carry out the standard 90° peeling tests on the adhered hydrogel samples under a single cycle and multiple cycles of loads to measure the interfacial toughness *Γ* (Fig. 2a) and interfacial fatigue threshold *Γ*_0_ (Fig. 2c), respectively. From a single-cycle peeling test, the interfacial toughness is measured as

1 $${\it{\Gamma }} = F_s/W,$$

where *F*_*s*_ is the steady-state peeling force and *W* is the width of the hydrogel sample (Fig. 2a)^19^. In a multiple-cycle peeling test, we apply the cyclic peeling force with an amplitude of *F*_*a*_ (*F*_*a*_ < *F*_*s*_) over *N* cycles, and measure the interfacial crack extension *c* as a function of cycle number *N*. Accordingly, the applied energy release rate *G* can be calculated as

2 $$G = F_a/W,$$

and the interfacial crack propagation rate as d*c*/d*N*. The multiple-cycle peeling test is performed at varied amplitudes of applied forces to give a plot of d*c*/d*N* versus *G* (Fig. 2c). By linearly extrapolating the plot of d*c*/d*N* versus *G* to intercept the *G*-axis, we can obtain the interfacial fatigue threshold *Γ*_0_, measured under this condition^20^ (Fig. 2d). Notably, the hydrogel samples are immersed in an aqueous bath and maintain their swollen state throughout the peeling tests to prevent dehydration. In addition, a thin and rigid nylon film is attached to the surface of the hydrogel sample as a backing to prevent its elongation along the peeling direction (see Methods for the details on the peeling tests).

As shown in Fig. 2b, while the measured interfacial toughnesses for both tough hydrogel adhesion (PAAm-alginate with 1500 J m^−2^) and fatigue-resistant hydrogel adhesion (PVA with 7500 J m^−2^, Fig. 2, Supplementary Fig. 2, and Supplementary Movie 1) on glass are high, their interfacial fatigue thresholds are dramatically different. The interfacial fatigue threshold of tough hydrogel adhesion (PAAm-alginate) is as low as 68 J m^−2^ (Fig. 2d and Supplementary Fig. 3), similar to that of common hydrogel adhesion (9 J m^−2^ for PAA and 32 J m^−2^ for PAAm, Supplementary Figs. 4 and 5; Table 1), and comparable with the energy required for fracturing a layer of amorphous polymer chains (1–100 J m^−2^)^2–4^. By contrast, the fatigue threshold of fatigue-resistant hydrogel adhesion is 800 J m^−2^ in both deionized water (Fig. 2d and Supplementary Fig. 6) and phosphate-buffered saline (Supplementary Figs. 7 and 8), approximating the value for tendon-to-bone adhesion^14–16^. To further validate the high interfacial fatigue threshold, we apply an energy release rate of 800 J m^−2^ on the fatigue-resistant hydrogel adhesion over 30,000 cycles, and observe no interfacial crack propagation using a camera with a resolution of 20 μm per pixel. This result means that the speed of any possible crack propagation should be lower than 0.6 nm/cycle, consistent with the previous validation of fatigue thresholds for hydrogels^17^ (Fig. 2f, Supplementary Fig. 9, and Supplementary Movie 2). On the contrary, when the tough hydrogel adhesion is subjected to an energy release rate of 200 J m^−2^ over 5000 cycles, substantial propagation of the interfacial cohesive crack can be identified (Fig. 2e).

Furthermore, when the applied energy release rate is higher than the interfacial fatigue threshold, the interfacial fatigue cracks in both cases (i.e., PAAm-alginate and PVA) may gradually tilt into the bulk hydrogel and induce fatigue fracture of the bulk hydrogel (Fig. 2e and Supplementary Fig. 10). This failure mode indicates that the interfacial fatigue thresholds of hydrogel adhesion also depend on the intrinsic fracture toughness of the bulk hydrogel, due to possible crack tilting and cohesive failure of the bulk hydrogel. The high density of nanocrystalline domains in the bulk PVA hydrogel gives it a high intrinsic fracture toughness (i.e., 1000 J m^−2^)^17^ and guarantees its high interfacial fatigue threshold (i.e., 800 J m^−2^). Moreover, the fatigue-resistant hydrogel adhesion exhibits long-term stability with consistent interfacial toughness and interfacial fatigue threshold after soaking in deionized water for 90 days (Supplementary Fig. 11).

In addition to glass, the fatigue-resistant hydrogel adhesion is also applicable to diverse solid substrates (Fig. 2g and Supplementary Fig. 12), including ceramics (*Γ*_0_ = 470 J m^−2^), titanium (225 J m^−2^), aluminum (370 J m^−2^), stainless W (330 J m^−2^), PU (420 J m^−2^), and PDMS (150 J m^−2^). Their interfacial fatigue thresholds are orders of magnitude higher than that of the corresponding tough hydrogel adhesion (Supplementary Figs. 13 and 14).

### Mechanism for fatigue-resistant hydrogel adhesion

To understand the mechanism for fatigue-resistant hydrogel adhesion, we vary the annealing time for the PVA hydrogels on glass substrates, and then measure the interfacial fatigue thresholds and characterize the nanocrystalline domains in the hydrogels. The hydrogel without annealing easily detaches from the substrates upon hydration. However, by increasing the annealing time from 0 to 90 min, the interfacial fatigue threshold of the hydrogel adhesion is greatly enhanced from 0 to 800 J m^−2^ (Supplementary Fig. 15). Correspondingly, the prolonged annealing time (from 0 to 90 min) leads to the significantly increased crystallinity (from 12.1 to 33.7 wt% at the swollen state) and reduced water content (from 67 to 37 wt%) in the PVA hydrogels (Supplementary Fig. 16). In addition, although the freeze–thawing process is crucial for nanocrystal nucleation in hydrogels, varying the freeze–thawing cycles of PVA hydrogels does not change the adhesion performance significantly (Supplementary Fig. 17), since the subsequent dry-annealing process increases the crystallinity much more dramatically than the freeze–thawing process^17^.

**Fig. 3:**
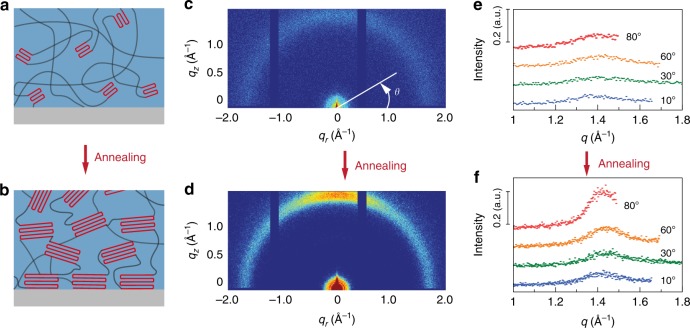
Mechanisms for fatigue-resistant hydrogel adhesion from experiments. **a**, **b** Schematic illustration of the nanocrystalline structures of fatigue-resistant hydrogel adhesion before (**a**) and after (**b**) annealing (100 °C, 90 min). **c**, **d** GIWAXS patterns of fatigue-resistant hydrogel adhesion before (**c**) and after annealing (**d**). **e**, **f** GIWAXS scattering profiles at azimuthal angles of 10°, 30°, 60°, and 80° for PVA hydrogel before (**e**) and after (**f**) annealing.

We further carry out two-dimensional (2D) grazing-incidence wide-angle X-ray scattering (GIWAXS) measurements on the PVA samples adhered to glass substrates before and after annealing (100 °C, 90 min). Before annealing, the 2D pattern of GIWAXS (Fig. 3c, e) exhibits a uniform scattering ring with equal intensity at all different azimuthal angles (*θ*), suggesting random orientation of the nanocrystalline domains (Fig. 3a)^21^. By contrast, an arc-like scattering feature prominently appears in the 2D pattern of GIWAXS for samples after annealing, where the intense scattering is dominantly aligned along the normal direction of the hydrogel–solid interface (Fig. 3d, f). This pattern implies that the preferred orientation of nanocrystalline domains is parallel to the interface in annealed PVA hydrogel samples (Fig. 3b)^22^. The densely packed and oriented nanocrystalline domains at the interface can collectively and effectively pin the crack propagation on the hydrogel–solid interface.

**Fig. 4:**
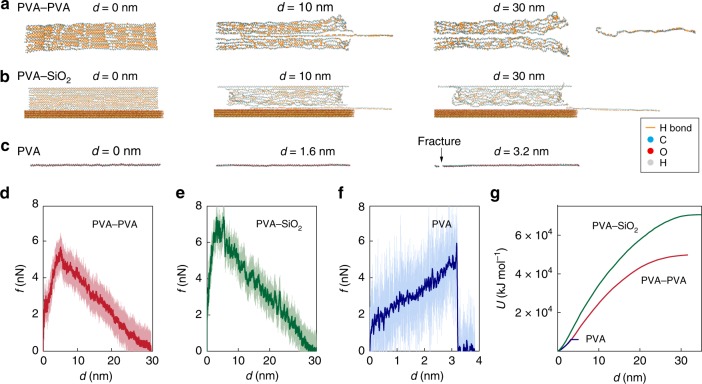
Mechanism for fatigue-resistant hydrogel adhesion from simulations. **a** An image sequence of pulling out a PVA polymer chain (30 nm in counter length) from a nanocrystalline domain (PVA–PVA) at different applied displacements *d* using molecular dynamics simulation. **b** An image sequence of pulling a PVA polymer chain (30 nm in counter length) from a nanocrystal-substrate interface (PVA–PVA) at different applied displacements *d*. Silica is used as a substrate. **c** An image sequence of pulling an amorphous PVA polymer chain (30 nm in counter length) (PVA) at different applied displacements *d* till fracture. **d**–**f** Force versus applied displacement for pulling out a PVA polymer chain from a nanocrystalline domain (PVA–PVA, **d**) pulling out a PVA polymer chain from a nanocrystal–substrate interface (PVA–SiO_2_, **e**), and pulling an amorphous PVA polymer chain till fracture (PVA, **f**). The lighter lines for the simulation results with force–displacement recorded every 200 fs and the solid lines for the moving average result with a window size of 20 ps and a pulling speed of 0.01 Å ps^−1^. **g** Strain energies versus applied displacement for pulling out a PVA polymer chain from a nanocrystalline domain (PVA–PVA), pulling out a PVA polymer chain from a nanocrystal–substrate interface (PVA–SiO_2_), and pulling an amorphous PVA polymer chain till fracture (PVA).

To quantify the effect of forming nanocrystalline domains on the interfacial fatigue threshold, we carry out all-atom molecular dynamics (MD) simulations to compare the energies required to pull out a PVA polymer chain (30 nm in contour length) from a nanocrystalline domain and to fracture an amorphous PVA polymer chain of the same contour length (see Supplementary Fig. 18 and Methods for detailed MD setup). It is shown in Fig. 4a and Supplementary Movie 3 that the PVA polymer chain is pulled out from the nanocrystalline domain in a stick-slip manner, because of the dynamic rupture and reforming of massive hydrogen bonds^23^. Despite the weak strength of a single hydrogen bond, multiple hydrogen bonds within a nanocrystal cooperatively sustain a maximal pull-out force of 6 nN (Fig. 4d, PVA–PVA), slightly higher than the force to fracture the amorphous PVA chain (5.8 nN, Fig. 4c, f, PVA). In addition, the pull-out process requires a displacement of ~30 nm (i.e., the contour length of the PVA chain), but the fracture process only requires a displacement of ~3.2 nm (i.e., ~10% of the contour length). Therefore, the cooperative rupture of hydrogen bonds demands a much higher strain energy (~50,000 kJ mol^−1^) to pull out the PVA chain than that to fracture an amorphous PVA chain with the same contour length (~6000 kJ mol^−1^). Moreover, pulling out of a PVA chain between the nanocrystal–glass interface requires a higher energy (~70,000 kJ mol^−1^, Fig. 4b, e, g, PVA–SiO_2_) than that out of a standalone nanocrystal (~50,000 kJ mol^−1^), which is consistent with our observation that the bulk hydrogel fractures during the peeling test, other than interfacial detachment (Supplementary Movie 1 and Supplementary Fig. 10). Therefore, introducing nanocrystalline domains on the interface and within the bulk hydrogel synergistically ensures a hydrogel–solid interface with extremely high fatigue resistance.

### Potential applications of fatigue-resistant hydrogel adhesion

**Fig. 5:**
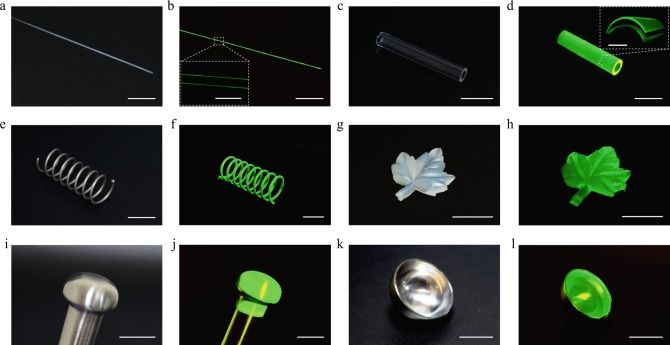
Fatigue-resistant hydrogel coatings on various materials with complex geometries. **a**, **b** A glass optical fiber (200 μm in diameter) with (**b**) and without (**a**) fatigue-resistant hydrogel coatings. Inset in **b** confocal laser-scanning microscopic (CLSM) image of the hydrogel coating (∽20 μm in thickness) on the glass optical fiber. **c**, **d** A glass tube with (**d**) and without (**c**) fatigue-resistant hydrogel coatings on the inner and outer surfaces. Inset in **d** 3D CLSM image of the hydrogel coating on both inner and outer surfaces. **e**, **f** A stainless steel spring with (**f**) and without (**e**) fatigue-resistant hydrogel coatings. **g**, **h** A leaf-shaped elastomer with (**h**) and without (**g**) fatigue-resistant hydrogel coatings. **i**, **j** A convex end of the ball-and-socket metallic joint with (**j**) and without (**i**) fatigue-resistant hydrogel. **k**, **l** A concave end of the ball-and-socket metallic joint with (**l**) and without (**k**) fatigue-resistant hydrogel coatings. The hydrogel coatings are colorized with fluorescein sodium salt dye for visualization and all the images of **b**, **d**, **f**, **h**, **j**, **l** are taken under a blue light excitation (480 nm in wavelength). Scar bars: 20 mm in (**a**–**l**), 500 μm in the inset of (**b**), and 2 mm in the inset of (**d**).

Materials and devices with complex shapes can be exposed to repeated mechanical loads, which pose a challenge to their coating materials in terms of fabrication method and long-term robustness. We demonstrate that the fatigue-resistant hydrogel adhesion can potentially provide a facile and versatile solution towards this challenge. We first present a set of materials and devices, including a glass optical fiber (Fig. 5a before and Fig. 5b after coating), a glass tube (Fig. 5c, d), a stainless steel spring (Fig. 5e, f), a leaf-shaped elastomer (Fig. 5g, h), and a ball-and-socket metallic joint (Fig. 5i-l), which are coated with a hydrogel layer through a dip-coating, freeze–thawing, and dry-annealing process (see Methods for details). The thin (~20 μm in thickness, insets in Fig. 5b, d) and uniform hydrogel coatings are applicable to devices with various feature sizes (from 200 μm in Fig. 5a to 35 mm in Fig. 5i), curvatures (convex surface in Fig. 5a, e, i, concave surface in Fig. 5k, and inner and outer surfaces in Fig. 5c) and diverse materials (glass in Fig. 5a, c, stainless steel in Fig. 5e, i, k, and silicone elastomer in Fig. 5g).

**Fig. 6:**
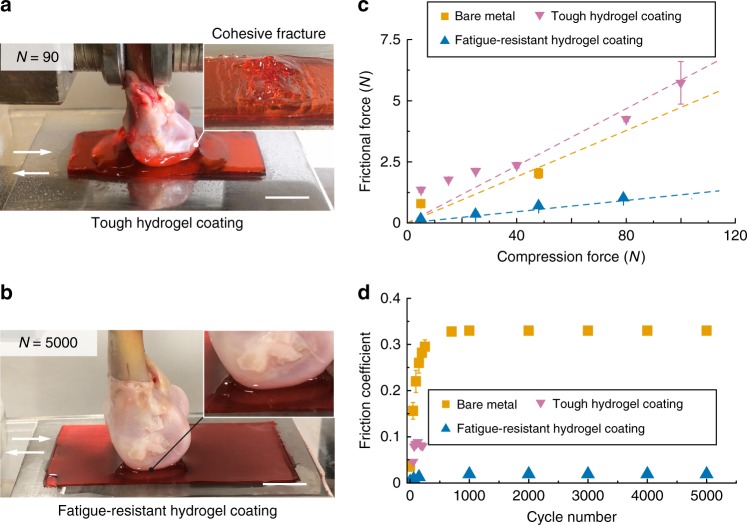
Fatigue-resistant hydrogel coating on a stainless steel substrate and sliding against natural cartilages. **a** Image of tough hydrogel coating on stainless steel plate against cartilage, showing cohesive fracture at the 90th cycle of reciprocating sliding (compression force of 100 N). **b** Image of fatigue-resistant hydrogel coating on stainless steel plate against cartilage, showing robust adhesion after 5000 cycles of reciprocating sliding (compression force of 100 N). **c** Frictional force versus normal compression force for various substrates against cartilages at the first cycle of reciprocating sliding (*N* = 1). **d** Evolution of friction coefficient over 5000 cycles of reciprocating sliding for various substrates against cartilages with a normal compression force of 100 N (compressive stress of ∽1 MPa) for bare metal and fatigue-resistant hydrogel coating, or a normal compression force of 20 N (compressive stress of ∽0.2 MPa) for tough hydrogel coating. Error bars = standard deviation (*n* = 3). Scale bars: 10 mm in (**a**, **b**).

Next, we adopt the ball-on-flat sliding test (ASTM G133, Supplementary Fig. 19a) to evaluate the adhesion, friction, and wear performances of our fatigue-resistant hydrogel coating on a stainless steel surface over cyclic mechanical loadings^24^. The PAAm–alginate hydrogel coating and the corresponding bare stainless steel are selected as control samples for comparison. As shown in Fig. 6a, b, a constant normal compression load (100 N) is applied on a chicken tibia cartilage (compressive stress of ~1 MPa), beneath which the sample for the sliding test undergoes reciprocating motion. Within 90 cycles, cohesive fracture occurs in the chemically anchored PAAm–alginate hydrogel coating (Fig. 6a and Supplementary Movie 4). On the contrary, the fatigue-resistant hydrogel coating remains mechanically robust and adheres on the substrate over 5000 cycles of reciprocating motion (Fig. 6b and Supplementary Movie 5).

We further measure the dynamic friction coefficients between cartilages and various samples under different compression forces and different cycles (Fig. 6c, d). In the first cycle, the measured friction coefficients for the fatigue-resistant hydrogel coating, PAAm–alginate hydrogel coating, and bare stainless steel surface are 0.006, 0.04, and 0.045, respectively. When the cycle number of the reciprocating motion reaches 90 (under a compression force of 20 N), the PAAm–alginate hydrogel coating fails as discussed above. As the cycle number increases from 1 to 200 (under a compression force of 100 N), the friction coefficient of the fatigue-resistant hydrogel coating increases from 0.006 to 0.02 and maintains around 0.02 until the end of the test (5000 cycles, Fig. 6d and Supplementary Fig. 20). Moreover, the friction coefficient of the bare metal surface increases from 0.045 to 0.3 as the cycle number increases from 1 to 300 (under a compression force of 100 N), and remain constant afterwards (Fig. 6d and Supplementary Fig. 20).

In addition, the abrasive wear properties on the cartilages against the fatigue-resistant hydrogel coating and the bare stainless steel surface after 5000 cycles of the reciprocating motion are compared in Supplementary Fig. 21. It is evident that the wear on the cartilage against the stainless steel surface is more severe than that against the fatigue-resistant hydrogel coating, which is consistent with the lower friction coefficient of the fatigue-resistant hydrogel coating. Furthermore, we do not observe significant wear on the surface of the fatigue-resistant hydrogel coating after 5000 cycles of the reciprocating motion using both confocal microscopy and scanning electron microscopy (Supplementary Fig. 22).

## Discussion

Inspired by the adhesion between bones and tendons/ligaments/cartilages in the human body, we develop a facile yet general strategy for the fatigue-resistant adhesion of hydrogels on diverse engineering materials through bonding ordered nanostructures in the hydrogels on the engineering materials. This hydrogel–solid adhesion achieves an interfacial fatigue threshold up to 800 J m^−2^, and enables applications such as long-lasting hydrogel coatings on devices with various feature sizes, curvatures, and substrate materials. In particular, as a hydrogel coating on metallic substrates, the fatigue-resistant hydrogel adhesion exhibits low friction and low wear under cyclic friction tests. As a versatile strategy for the design of fatigue-resistant soft-hard interfaces, it is applicable to a wide range of hydrogel materials (e.g., nanofibrillar hydrogels and nanocomposite hydrogels) and solid substrates (e.g., metals, ceramics, and elastomers). The reported strategy for achieving fatigue-resistant adhesion of synthetic hydrogels to various engineering materials makes a number of future research directions and applications possible by providing a soft cushion between rigid machines and human body. For example, as a mechanically and biologically compatible interface, the fatigue-resistant hydrogel coating on machines can not only transmit electrical, optical, acoustic, chemical and mechanical signals between tissues and external devices (e.g., electrodes, optical fibers, ultrasound transducers, prosthetics, and robots), but also show robust performances over the long term.

## Methods

### Materials

For fatigue-resistant hydrogel adhesion, PVA powders (Mw 146–186 kDa, 99+% hydrolyzed; Sigma-Aldrich 363065) were used. For tough hydrogel adhesion, acrylamide (AAm; Sigma-Aldrich A8887) was used as the monomer, *N, N’*-methylenebisacrylamide (MBAA; Sigma-Aldrich 146072) as cross-linker, ammonium persulphate (APS; Sigma-Aldrich A3678) as thermal initiator and *N, N, N’, N’*-tetramethylethylenediamine (TEMED; Sigma-Aldrich T9281) as crosslinking accelerator, and alginate (Sigma-Aldrich A2033) ionically cross-linked with calcium sulfate (Sigma- Aldrich C3771) as the dissipative polymer network. AAm and acrylic acid (AA; Sigma-Aldrich 147230) were used as monomers for two types of brittle hydrogel adhesion. The functional silane solution containing 500 mL of deionized water, 50 μL of acetic acid (Sigma-Aldrich 27225), and 2 mL of 3-(trimethoxysilyl) propyl methacrylate (TMSPMA; Sigma-Aldrich 440159) was used for chemical modification of substrates before forming tough hydrogel adhesion. Food dyes (red, green, and blue, McCormick) and fluorescein sodium salt (Sigma-Aldrich F6377) were added in the hydrogels for visualization and fluorescent labeling, respectively. Dulbecco’s phosphate-buffered saline (DPBS; Gibco) and water were used as media for peeling test.

Borosilicate glass (McMaster Carr, 8476K36), nonporous glass mica ceramic (McMaster Carr, 8489K232), titanium (McMaster Carr, 9039K15), anodized aluminum (McMaster Carr, 89015K181), stainless steel (McMaster Carr, 8983K115), PDMS (Dow Corning, Sylgard 184), Ecoflex (Smooth-On, 00-10), and PU (Smooth-On, Smooth-Cast 300) were adopted as various solid substrates for hydrogel adhesion. A nylon film (100 µm in thickness; TISCH, RS10133) was used as a stiff backing for the hydrogel samples in the peeling test. Glass optical fiber (200 μm in diameter, with coating and cladding layer removed; Thorlabs, FG200UEA), borosilicate glass tube (McMaster Carr, 8729K66), corrosion-resistant compression spring (McMaster Carr, 9663K88), stainless steel mortar pestle (Bekith, demo as metallic convex shape), stainless steel measuring spoon head (Amazon, demo as metallic convex shape) were used for the dip coating.

The simulated synovial fluid was prepared following the recipe in the previous literature^25^. 0.5 wt% hyaluronic acid (HA, *M*_w_ 750–1000 kDa; Sigma-Aldrich, 9067-32-7), 0.01 wt% *L*-*α*-dipalmitoyl phosphatidylcholine (DPPC; Sigma-Aldrich, 200-567-6) as liposomes, 1.4 wt% albumin (Sigma-Aldrich, 232-936-2), and 0.7 wt% *γ*-globulin (Sigma-Aldrich, 232-706-1) as protein additives were dissolved in DPBS.

### Fatigue-resistant hydrogel adhesion to various substrates

10 g of PVA powders were dispersed in 90 mL deionized water, and dissolved at 100 °C for 5 h, followed by defoaming with a centrifugal mixer (AR-100, Thinky). All the substrates (i.e., glass, ceramic, titanium, aluminum, stainless steel, PDMS, and PU) were thoroughly cleaned with ethanol and deionized water, and then completely dried with nitrogen flow. For the stainless steel substrates, they were first polished to remove the surface oxidation layer prior to the cleaning. Cleaned substrates were then treated by oxygen plasma (30 W at a pressure of 200 mTorr, Harrick Plasma PDC-001) for 5 min. Immediately after the plasma treatment, the PVA solution was poured onto the substrate within an acrylic spacer (Plexiglass, 45 mm × 20 mm × 1.5 mm), fully covered with an acrylic plate (Plexiglass, 75 mm × 50 mm × 1.5 mm), frozen at −20 °C for 8 h, and thawed at 25 °C for 3 h. Multiple cycles of freeze–thawing (cycle number = 1 – 5) could be done, but unless otherwise specified, we only performed one freeze–thawing cycle. Then, the acrylic cover and spacer were removed, and the freeze–thawed hydrogels were air-dried on the substrate for 8 h and then annealed at 100 °C for 90 min to obtain the fatigue-resistant adhesion between PVA hydrogels and substrates. The annealing time was varied when investigating the effect of annealing time on the PVA hydrogel adhesion (Supplementary Fig. 15). The Young’s modulus of PVA hydrogels increases from 200 kPa to 10 MPa as the annealing time increases from 0 to 90 min^17^. All PVA hydrogels adhered to diverse substrates were immersed in water to reach the swollen state before testing.

### Tough hydrogel adhesion to various substrates

The tough hydrogel adhesion to various substrates was fabricated following the previous protocol^1^. Briefly, the surface of substrates (i.e., glass, ceramics, titanium, aluminum, and stainless steel) was functionalized with TMSPMA by incubating the substrates in the functional silane solution for 2 h at room temperature. Substrates were washed with ethanol and completely dried with nitrogen flow. The PAAm–alginate tough hydrogel adhesion was fabricated by mixing a carefully degassed aqueous precursor solution (12 wt% AAm, 2 wt% sodium alginate, 0.023 wt% MBAA, and 0.043 wt% APS) with calcium sulfate slurry (0.27 wt% in the mixture) and TEMED (0.1 wt% in the mixture). The mixture was poured onto the TMSPMA-functionalized substrate within an acrylic frame (Plexiglass, 45 mm × 20 mm × 1.5 mm), covered with a glass slide (75 mm × 25 mm × 1 mm), and incubated at 50 °C for 1 h to achieve tough hydrogel adhesion. The Young’s modulus of the tough hydrogel is 40 kPa^26^. The tough hydrogels adhered to diverse substrates were immersed in water to reach the swollen state before testing.

### Brittle hydrogel adhesion to glass

Following the same surface functionalization as tough hydrogel adhesion, PAA precursor solution (20 wt% AA, 0.023 wt% MBAA, and 0.043 wt% APS) and PAAm precursor solution (20 wt% AAm, 0.023 wt% MBAA, 0.043 wt% APS, and 0.1 wt% TEMED) were poured onto the TMSPMA-functionalized substrate within an acrylic frame (Plexiglass, 45 mm × 20 mm × 1.5 mm), covered with a glass slide (75 mm × 25 mm × 1 mm), and incubated at 50 °C for 1 h to achieve hydrogel adhesion. The brittle hydrogel adhesions were immersed in water to reach the swollen state before testing.

### Measurement of interfacial toughness and interfacial fatigue threshold

The interfacial toughness of various hydrogel adhesions was measured using the standard 90° peeling test (ASTM D2861) with a mechanical testing machine (2 kN load cell; Zwick/Roell Z2.5) and 90° peeling fixture (TestResources, G50). As a rigid backing during peeling, a nylon film was bonded on the top of hydrogel with superglue (Loctite, 1405419). All 90° peeling tests were performed on swollen hydrogels immersed in a deionized water bath or DPBS at a constant loading speed of 120 mm min^−1^. The interfacial toughness was measured as *Γ* = *F*_*s*_/*W*, where *F*_*s*_ is the steady-state peeling force and *W* is the width of the hydrogel sample^19^.

To quantify the interfacial fatigue threshold of the hydrogel adhesion to various substrates, we further performed the 90° peeling test under cyclic loading (force control mode) using the same experiment setup as the standard 90° peeling test. In a multiple-cycle peeling test, we applied the cyclic peeling force with an amplitude of *F*_*a*_ (*F*_*a*_ < *F*_*s*_) over *N* cycles, and the interfacial crack extension *c* were recorded from the testing machine as a function of cycle number *N*. Accordingly, the applied energy release rate was calculated as *G* = *F*_a_/*W*, and the interfacial crack propagation rate as d*c*/d*N*. The multiple-cycle peeling test was performed at varied applied force amplitudes to give a plot of d*c*/d*N* versus *G*. By linearly extrapolating the plot of d*c*/d*N* versus *G* to the *x*-intercept, we obtained the interfacial fatigue threshold *Γ*_0_, measured under this condition^20^.

### Fatigue-resistant hydrogel coatings

The devices, including glass optical fiber, glass tube, stainless steel spring, Ecoflex elastomer, and ball-and-socket metallic joints, were cleaned with ethanol and deionized water, dried with nitrogen flow, and treated by oxygen plasma for 5 min. Immediately after the plasma treatment, the devices were dipped in the 10 wt% PVA solution and hung in a sealed container at room temperature and relative humidity of 90% for 1 h to drain excess PVA solution. The subsequent freeze–thawing, dry-annealing, and swelling were conducted following the above-mentioned procedure.

To visualize the PVA hydrogel coating on diverse substrates and shapes, 0.01 wt% of fluorescein sodium salt for fluorescent labeling was added into the PVA solution prior to dip-coating. The obtained PVA hydrogel-coated substrates were rinsed with deionized water to remove excess dye solution from the surface before imaging. Macroscopic images were taken by a digital camera (D7000, Nikon) under blue light excitation at 480 nm. The hydrogel coating was also imaged using an upright confocal laser-scanning microscope (CLSM; Leica TCS SP8), where the laser intensity, filter sensitivity, and grayscale threshold were adjusted in each application to optimize the contrast of images. A z-stack acquisition program (~20 µm in slice thickness, 50 steps) was used for 3D scanning, and 3D reconstruction images were created using the LAS X 3D visualization software.

### Friction test

We adopted the ball-on-flat sliding test (ASTM G133)^24^, which had also been used to evaluate the wear property of hydrogel coatings^27^. Fresh chicken tibia cartilages were purchased from a local butcher shop. All procedures were conducted in accordance with protocols approved by the Massachusetts Institute of Technology Committee on Animal Care. A constant normal compression load was applied on a chicken tibia cartilage, beneath which a substrate underwent a reciprocating sliding motion. Substrates included bare stainless steel, PVA hydrogel adhered to stainless steel, tough hydrogel adhered to stainless steel. The surface of each substrate was sprayed with a thin layer of simulated synovial fluid as a lubricant to mimic the working environment of knee joints. We also installed bearings beneath the substrate to reduce friction from the supporting structure (Supplementary Fig. 19). The normal compression load that applied on the chicken tibia cartilage by a mechanical testing machine (Zwick/Roell Z2.5) was 5, 20, 25, 45, 80, and 100 N. The velocity of reciprocating sliding was set at 5 mm s^−1^, and the amplitude of sliding distance as 10 mm. We recorded the frictional force between the substrate and cartilage continuously through a force logger (NUL-211, Neulog Sensors). The friction coefficients were calculated by dividing the averaged frictional force by the normal compression load.

### Wear test

Following the same procedure of friction test, we applied the normal compression load of 100 N on a chicken tibia cartilage, while the substrate underwent the reciprocating sliding motion for 5000 cycles. The compressive stress between cartilage and substrate was ~1 MPa, similar to the compressive stress in human knee joint^27^. Once the 5000-cycle test was completed, the cartilage was soaked in DPBS for 10 min prior to imaging with a digital camera (D7000, Nikon). Slices of cartilage in contact area were sampled and stained with fluorescein for further confocal laser-scanning microscopy. For the 3D scanning, z-stack acquisition program (~500 µm in slice thickness, 100 steps) was used, and a 3D reconstruction image was created using the LAS X 3D visualization software. The surface morphology of hydrogel coating before and after cyclic loads was imaged via a scanning electron microscope (JEOL 6010LA).

### Grazing-incidence wide-angle X-ray scattering (GIWAXS) measurement

Samples for GIWAXS measurements were prepared by spin-coating (3000 rpm, 45 s) of a PVA solution (10 wt%) on a clean glass slide (1 cm × 1 cm), freeze–thawing of the thin hydrogel layer in a humidified chamber and dry-annealing, resulting in a dry film of PVA (~300 nm in thickness) on a glass substrate for GIWAXS measurement. On the other hand, a PVA dry film on a glass substrate without annealing, and a PVA dry film annealed on a polytetrafluoroethylene substrate were used as controls (Supplementary Figs. 23 and 24). GIWAXS patterns of the PVA samples were obtained with a Pilatus3R 300K detector (Bruker Nanostar), at a radiation wavelength of 1.157 Å and bandwidth of 1.5%. The angle of incidence was kept constant at ~0.2°, while the reflection angle 2*θ* was scanned from 3 to 90° in angular steps of 0.1°.

### Molecular modeling

MD simulation of PVA crystal and its deformation under mechanical loading force were simulated by using the modified CHARMM27 force field implemented by using CHARMM General Force (CGFF) field for PVA model and a formerly developed force field for silica–water interface^28^. All the parameters for the modeling of PVA by CGFF yield the highest penalty of 0.2, which is significantly lower than 10 and strongly indicates the modeling is correct^29^. We also validated the geometry of PVA by using discrete Fourier transform calculation with the 6–31G basis set and geometric optimization with the conjugate gradient method and found good agreement with the current force field. This modified force field enables the calculation of a PVA–water–silica hybrid system. We ran MD simulation with NAMD package v2.13^30^. Our nanocrystal model starts from a structure with fully extended chains in parallel with 4.1 Å distance, in analogy to the parallel beta-sheet structures in proteins^23^. It is noted that this conformation (Fig. 4a; Supplementary Fig. 18) agrees with the former experimental characterization for the spatial distribution of hydroxyl groups^31^. We added a solvent box around the PVA structure with TIP3P water model covering at least 10 Å from the protein structure. The net charge of the system is zero by adding NaCl of the overall concentration of 0.1 mol L^−1^ and adjust the ratio of ions to neutralize the system.

### MD simulation

All the nanocrystalline structures were equilibrated in an NPT ensemble at constant temperature (300 K) and constant pressure (1 atmosphere) controlled by Langevin thermostat and barostat. The simulation time step is 2 fs with rigid bonds model for all the covalent bonds between hydrogen atoms and other heavy atoms. We used particle mesh Ewald function with a grid width <1 Å to calculate the electronic interaction because and it is an efficient method to accurately include all the long-distance electrostatics interactions. A cutoff length of 10 Å was applied to the van der Waals interactions and to switch the electrostatic forces from short-range to long-range forces. We followed the following protocol to equilibrate the structure: (1) energy minimization (10,000 conjugate gradient steps); (2) equilibration runs for 20 ns (NPT ensemble), where the temperature stays at 300 K. The carbon atoms of the unit at the right end of the middle PVA chain were pulled by using steered MDs, while the carbon atoms of the units at the other end of the rest chains were fixed. The pulling force was recorded versus the position. The simulations were carried out at pulling velocities of 0.01 Å ps^−1^ until the chain was fully pulled out from the nanocrystal.

For the pulling test of a single chain until the rupture of covalent bonds, we fixed the carbon atoms at the left end of the chain and pulled the carbon atoms at the right end at the same speed of 0.01 Å ps^−1^. It is noted that the CHARMM force field does not include rupture of covalent bonds and we thus used first-principles based ReaxFF force field^32^ to evaluate the mechanical behavior of the PVA chain until failure.

### Reporting summary

Further information on research design is available in the Nature Research Reporting Summary linked to this article.

## Supplementary information

Supplementary Information

Description of Additional Supplementary Files

Supplementary Movie 1

Supplementary Movie 2

Supplementary Movie 3

Supplementary Movie 4

Supplementary Movie 5

Reporting Summary

## Data Availability

The data that support the findings of this study are available from the corresponding author upon request.
