# Supplementary material for: Fatigue-resistant adhesion of hydrogels
Source: Nat Commun. 2020 Feb 26;11:1071. doi: 10.1038/s41467-020-14871-3 (PMC7044439; doi:10.1038/s41467-020-14871-3)
Supplement: Supplementary file 1 — Supplementary Information [file 41467_2020_14871_MOESM1_ESM.pdf]

Supplementary Information for

Fatigue-resistant adhesion of hydrogels

Liu et al.

Supplementary Note 1

Measurement of water content and crystallinities. The water content within the PVA hydrogels was quantified following the previous protocol[1]. Specifically, the swollen hydrogel samples scratched from the substrates weighing m_{swollen} were incubated at 37 °C for 2 h and weighed m after air-drying. The mass of the residual water in the as-prepared dry samples m_{residual} was measured in the previous section. Therefore, the water content in the swollen state can be calculated as $(m_{\text{swollen}} - m + m_{\text{residual}})/m_{\text{swollen}}$, and the polymer content in the swollen state can be calculated as $(m - m_{\text{residual}})/m_{\text{swollen}}$.

We measured the crystallinities of the resultant PVA hydrogels by differential scanning calorimetry (DSC/cell: RCS1-3277, cooling system: DSC1-0107), following the experimental protocols in our previous report[2]. PVA hydrogel adhered to the substrates were carefully scratch off with a razor blade, and dried at 37 °C for 2 h.

In a typical DSC measurement, we first weighed the total mass of the air-dried sample m (still with residual water). The sample was thereafter placed in a Tzero pan and heated up from 50 °C to 250 °C at the rate of 20 °C min^{-1} under a nitrogen atmosphere with a flow rate of 30 mL/min. The curve of heat flow shows a broad peak from 60 °C to 180 °C, indicating that the air-dried sample contained a small amount of residual water. The integration of the endothermic transition ranging from 60 °C to 180 °C gives the enthalpy for evaporation of the residual water per unit mass of the dry sample (with residual water) $H_{residual}$. Therefore, the mass of the residual water $m_{residual}$ can be calculated as $m_{residual} = m \cdot H_{residual} / H^0_{water}$, where $H^0_{water} = 2260$ J/g is the latent heat of water evaporation. The curve of heat flow shows another narrow peak ranging from 200 °C to 250 °C corresponding to the melting temperature of the crystalline domains. The integration of the endothermic transition ranging from 200 °C to 250 °C gives the enthalpy for melting the crystalline domains per unit mass of the dry sample (with residual water) $H_{crystalline}$. Therefore, the mass of the crystalline domains $m_{crystalline}$ can be

calculated as $m_{crystalline} = m \cdot H_{crystalline} / H^0_{crystalline}$, where $H^0_{crystalline} = 138.6$ J/g is the enthalpy of fusion of 100 wt.% crystalline PVA measured at the equilibrium melting point T_m^0 [3]. Therefore, the crystallinity in the ideally dry sample X_{dry} (without residual water) can be calculated as $X_{dry} = m_{crystalline} / \left(m - m_{residual} \right)$. With measured water content, the crystallinity in the swollen state can be calculated as $X_{swollen} = X_{dry} \cdot \left(1 - \phi_{water} \right)$.

Supplementary Table 1. Summary of various hydrogel adhesions on glass.

Hydrogel	Interfacial toughness (J m^{-2})	Interfacial fatigue threshold (J m^{-2})
PAA	300	9
PAAm	150	32
PAAm-alginate	1500	68
PVA (90-min annealing)	7000	800

Supplementary Figures

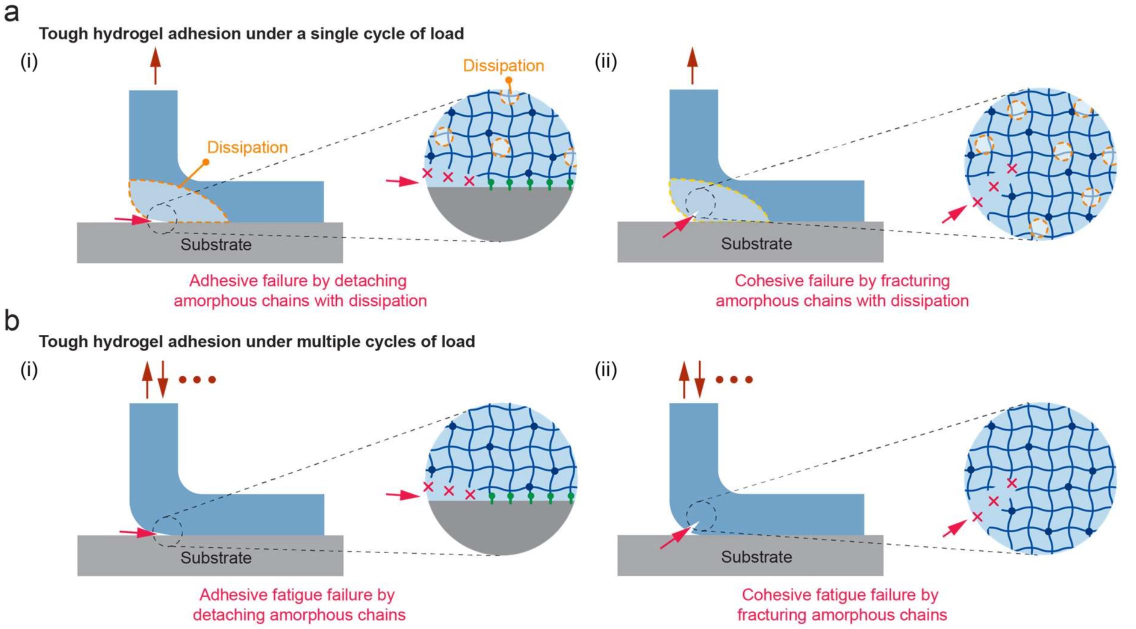

Supplementary Figure 1. **Failure modes in existing tough hydrogel adhesion. a,** Schematic illustration of the failure modes in tough hydrogel adhesion during a single cycle of loading: (**a-i**) adhesive failure by detaching the chemically-anchored amorphous chains from the substrates and (**a-ii**) cohesive failure by fracturing the amorphous chains, accompanied with substantial energy dissipation within the bulk hydrogel. **b,** Schematic illustration of the fatigue-induced failure modes in tough hydrogel adhesion under multiple cycles of loading: (**b-i**) adhesive failure by detaching the amorphous chains from the substrates and (**b-ii**) cohesive failure by fracturing the amorphous chains, with all energy dissipation depleted in both fatigue-induced failure modes. The resistance to fatigue crack propagation after prolonged cycles of loading in tough hydrogel adhesion is the energy required to fracture a single layer of polymer chains (i.e., intrinsic fracture energy of the hydrogel), which is unaffected by the additional dissipation mechanisms.

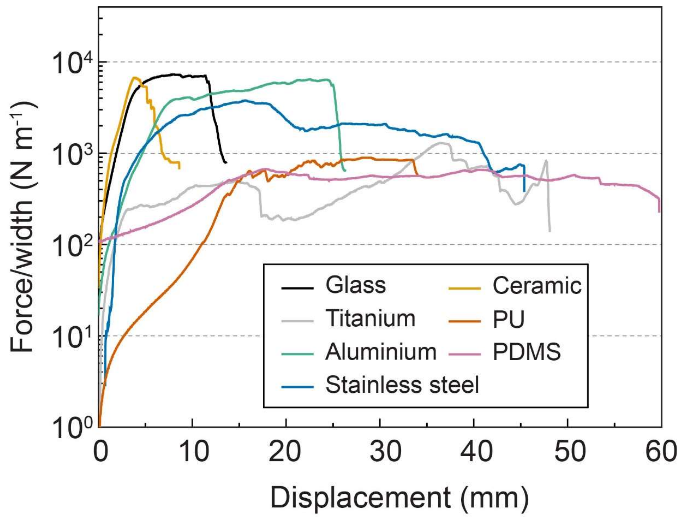

Supplementary Figure 2. **Interfacial toughness of fatigue-resistant hydrogel adhesion on diverse substrates.** Peeling curves for the fatigue-resistant hydrogel adhesion on diverse solids, including glass, ceramics, stainless steel, aluminium, titanium, PDMS and PU. A peeling rate of 120 mm min^{-2} was used.

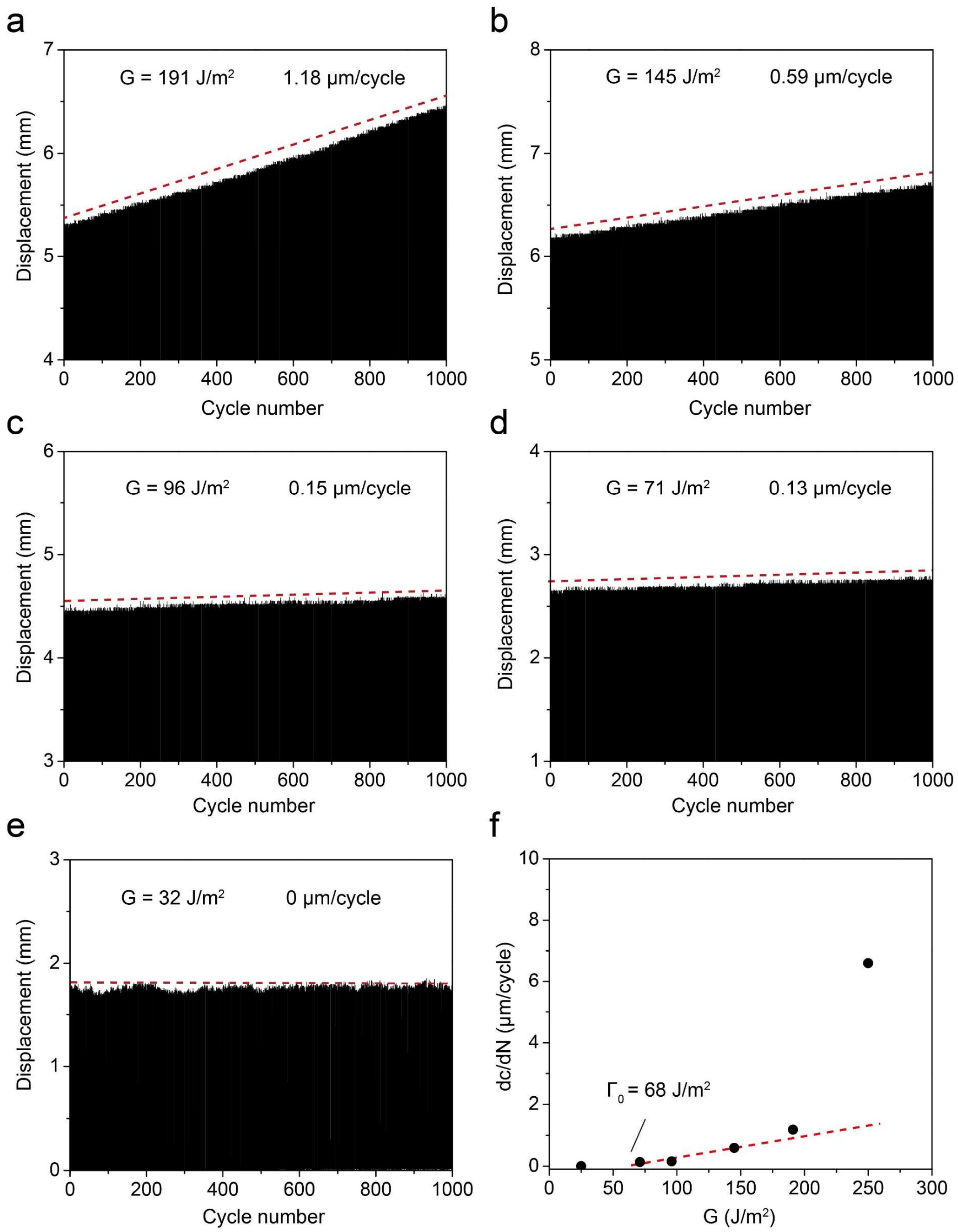

Supplementary Figure 3. Measurement of interfacial fatigue threshold of tough hydrogel adhesion to glass. Measured crack extension per cycle at applied energy release rates of **a.** 191 J m^{-2}, **b.** 145 J m^{-2}, **c.** 96 J m^{-2}, **d.** 71 J m^{-2} and **e.** 32 J m^{-2}. **f.** Measured crack extension per cycle (dc/dN) versus applied energy release rate G. The interfacial fatigue threshold of tough hydrogel adhesion (i.e., PAAm-alginate) on glass is measured to be 68 J m^{-2} by linearly extrapolating the curve to intercept with the abscissa.

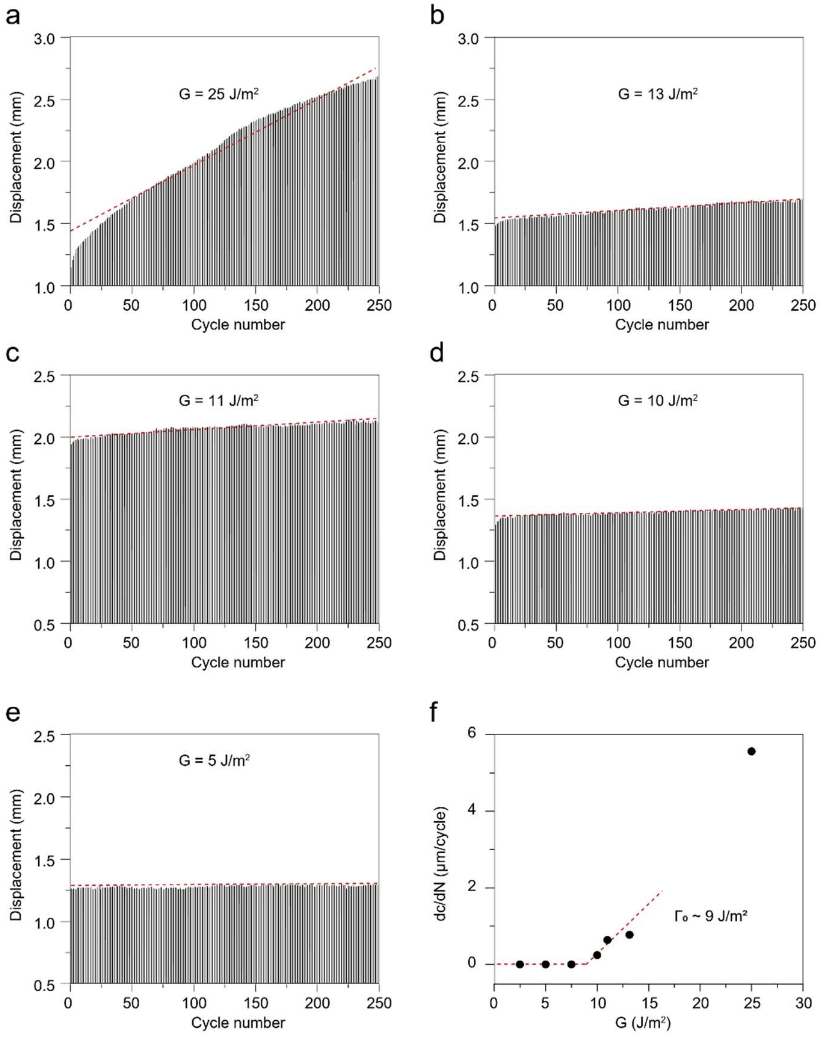

Supplementary Figure 4. Measurement of interfacial fatigue threshold of common hydrogel (PAA) adhesion to glass. Measured crack extension per cycle at applied energy release rates of **a.** 25 J m^{-2}, **b.** 13 J m^{-2}, **c.** 11 J m^{-2}, **d.** 10 J m^{-2} and **e.** 5 J m^{-2}. **f.** Measured crack extension per cycle (d*c*/d*N*) versus applied energy release rate *G*. The interfacial fatigue threshold of brittle hydrogel adhesion (i.e., PAA) on glass is measured to be 9 J m^{-2} by linearly extrapolating the curve to intercept with the *G-axis*.

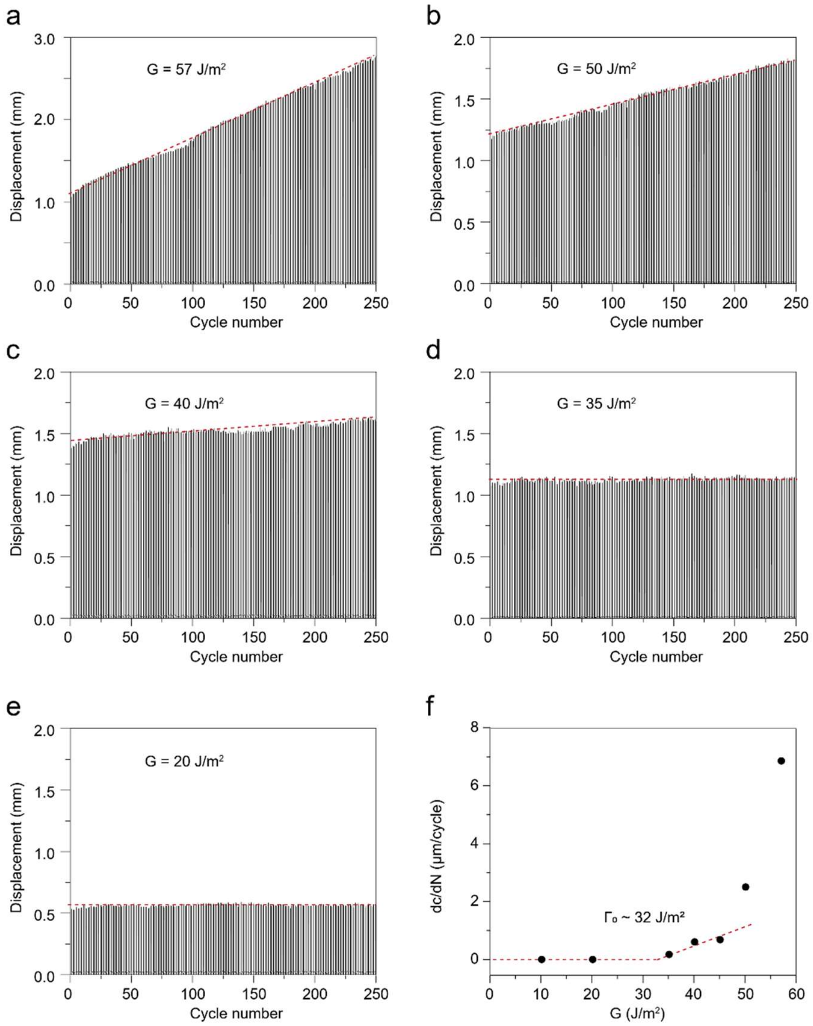

Supplementary Figure 5. Measurement of interfacial fatigue threshold of common hydrogel (PAAm) adhesion to glass. Measured crack extension per cycle at applied energy release rates of **a.** 57 J m^{-2}, **b.** 50 J m^{-2}, **c.** 40 J m^{-2}, **d.** 35 J m^{-2} and **e.** 20 J m^{-2}. **f.** Measured crack extension per cycle (dc/dN) versus applied energy release rate G. The interfacial fatigue threshold of brittle hydrogel adhesion (i.e., PAAm) on glass is measured to be 32 J m^{-2} by linearly extrapolating the curve to intercept with the G-axis.

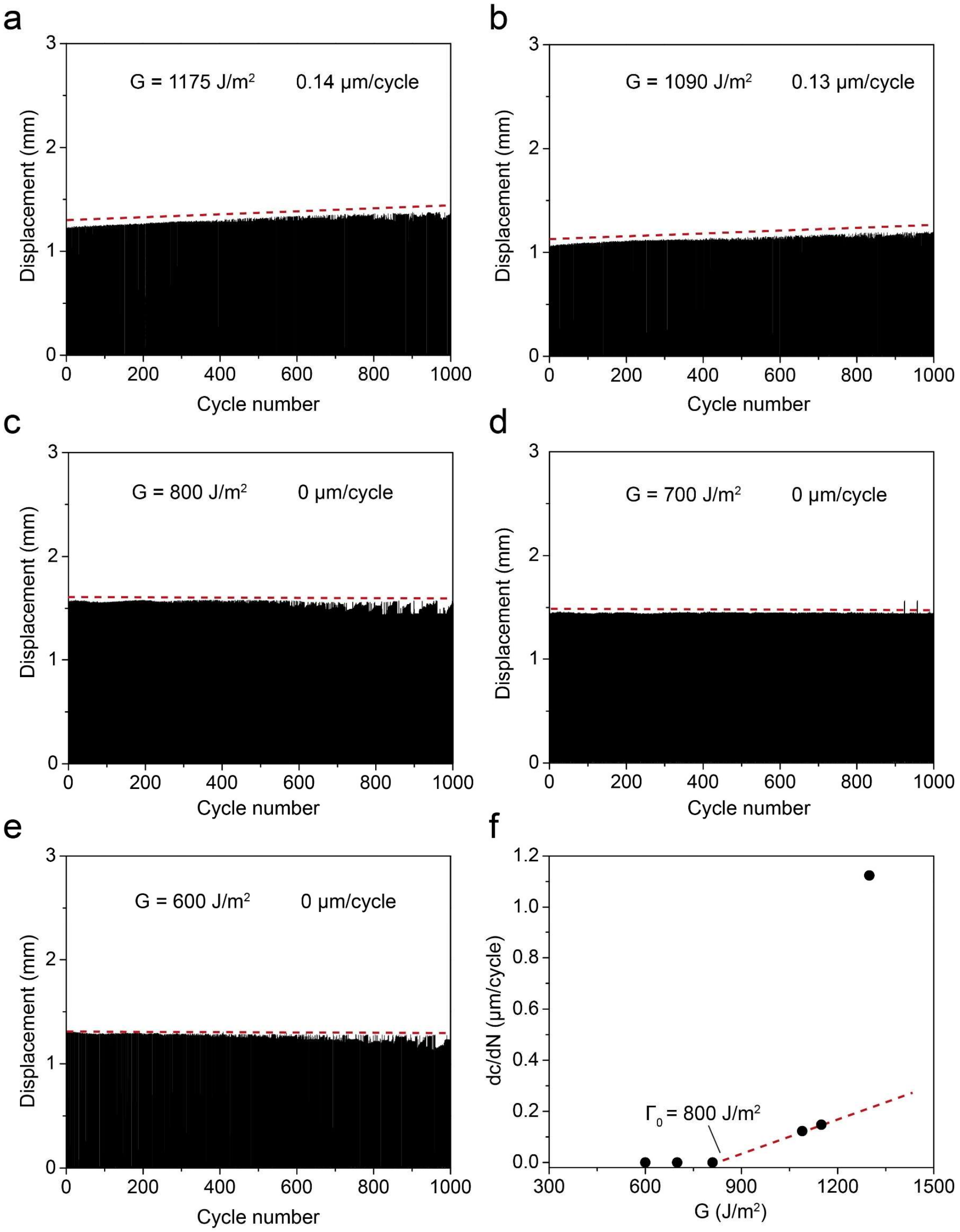

Supplementary Figure 6. **Measurement of interfacial fatigue threshold of fatigue-resistant hydrogel adhesion to glass.** Measured crack extension per cycle at applied energy release rates of **a.** 1,175 J m^{-2}, **b.** 1,090 J m^{-2}, **c.** 800 J m^{-2}, **d.** 700 J m^{-2} and **e.** 600 J m^{-2}. **f.** Measured crack extension curve in the plot of crack extension per cycle (d*c*/d*N*) versus applied energy release rate *G*. The interfacial fatigue threshold of fatigue resistant hydrogel adhesion to glass is measured to be 800 J m^{-2} by linearly extrapolating the curve to intercept with the *G*-axis.

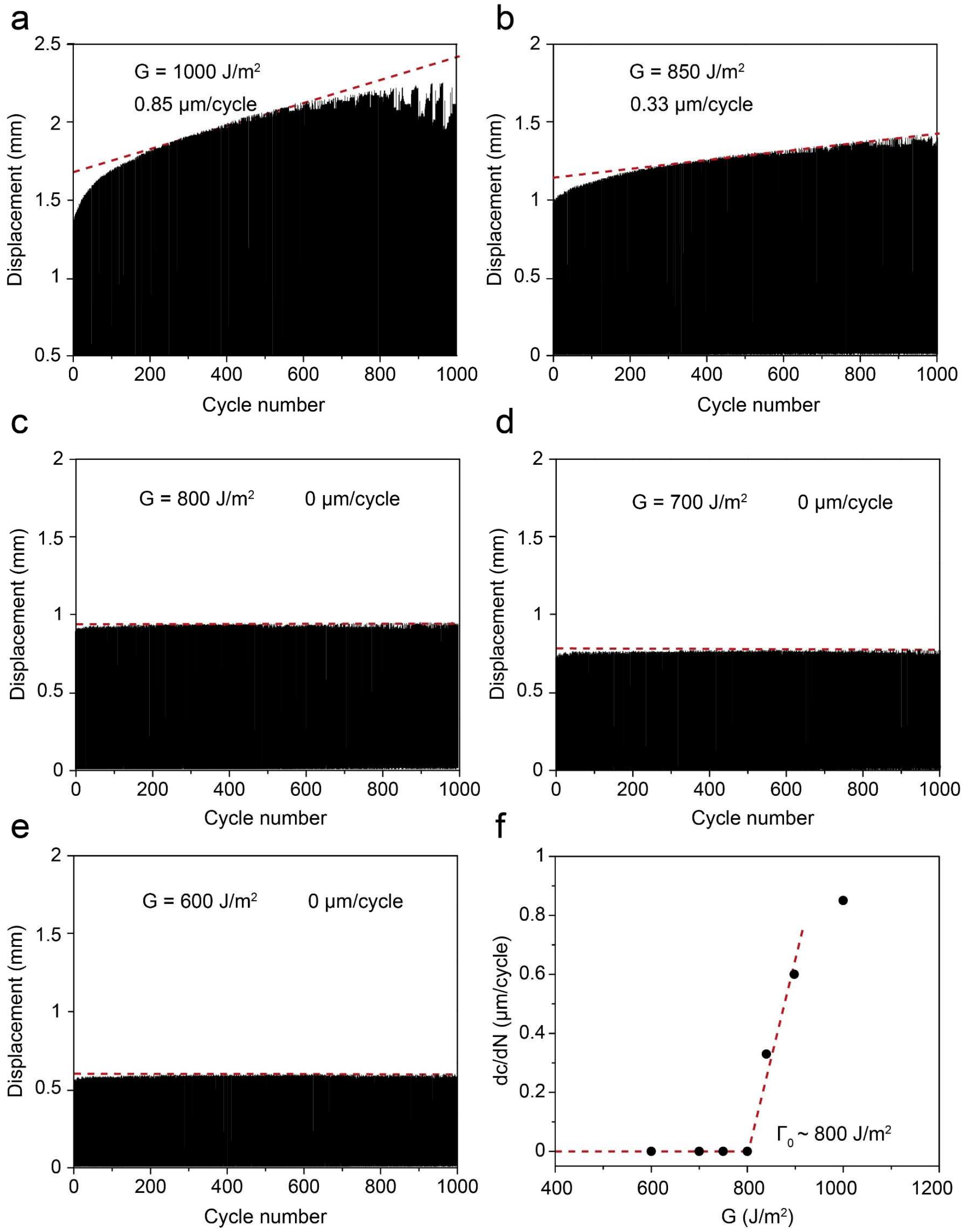

Supplementary Figure 7. **Measurement of interfacial fatigue threshold of fatigue-resistant hydrogel adhesion to glass in Dulbecco's phosphate-buffered saline.** Measured crack extension per cycle at applied energy release rates of **a.** 1,000 J m^{-2}, **b.** 850 J m^{-2}, **c.** 800 J m^{-2}, **d.** 700 J m^{-2} and **e.** 600 J m^{-2}. **f.** Measured crack extension curve in the plot of crack extension per cycle (dc/dN) versus applied energy release rate G. The interfacial fatigue threshold of fatigue resistant hydrogel adhesion to glass is measured to be 800 J m^{-2} by linearly extrapolating the curve to intercept with the G-axis.

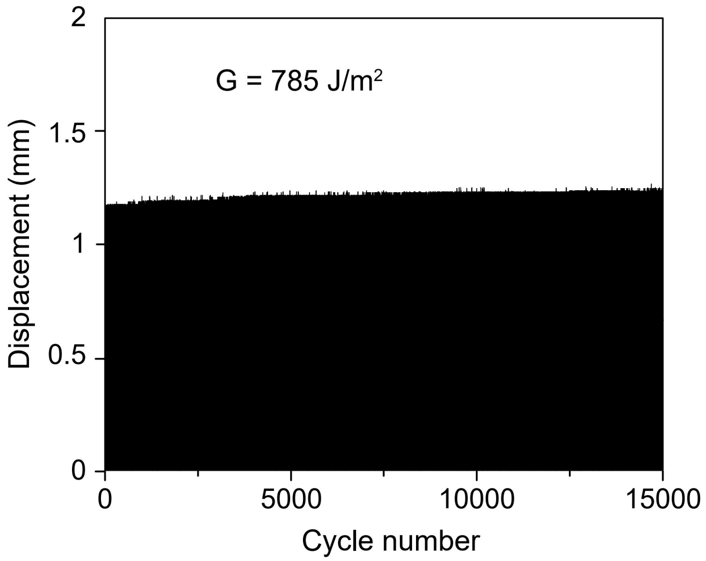

Supplementary Figure 8. Validation of interfacial fatigue threshold. The interfacial fatigue threshold of fatigue-resistant hydrogel adhesion is validated as high as 785 J/m^2 in PBS over 15,000 cycles of peeling test.

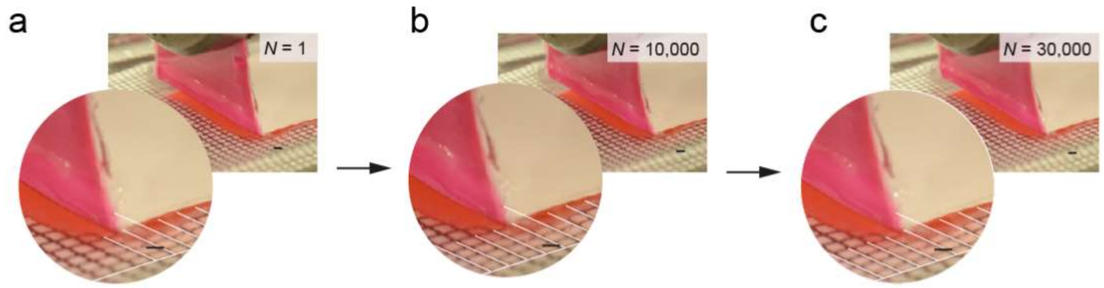

Supplementary Figure 9. **Validation of high fatigue threshold of the PVA hydrogel adhesion on a glass substrate.** Images of PVA hydrogel adhered on glass at an applied energy release rate of 800 J m^{-2} at the cycle number of 10,000, 20,000, and 30,000. No interfacial crack propagation has been observed using a camera with a resolution of 20 μm per pixel. Scale bars: 1 mm.

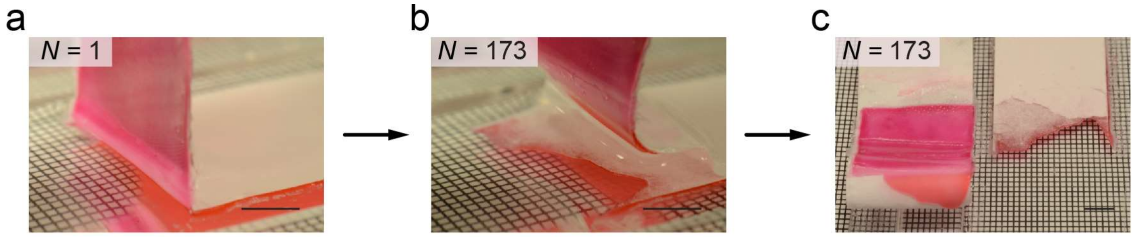

Supplementary Figure 10. Fatigue failure mode of fatigue-resistant hydrogel adhesion under the peeling test. The fatigue crack tilts on the interface and propagates into the bulk hydrogel, accompanied by the rupture of both hydrogel and backing at an energy release rate of 1,500 J m^{-2} within 173 cycles. Scale bars: 5 mm.

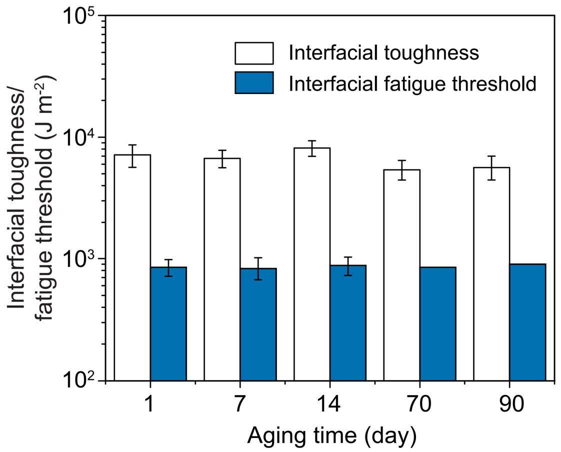

Supplementary Figure 11. Aging properties of the fatigue-resistant hydrogel adhesion to glass. Summary of the interfacial toughness and interfacial fatigue threshold versus the aging time when soaking in deionized water.

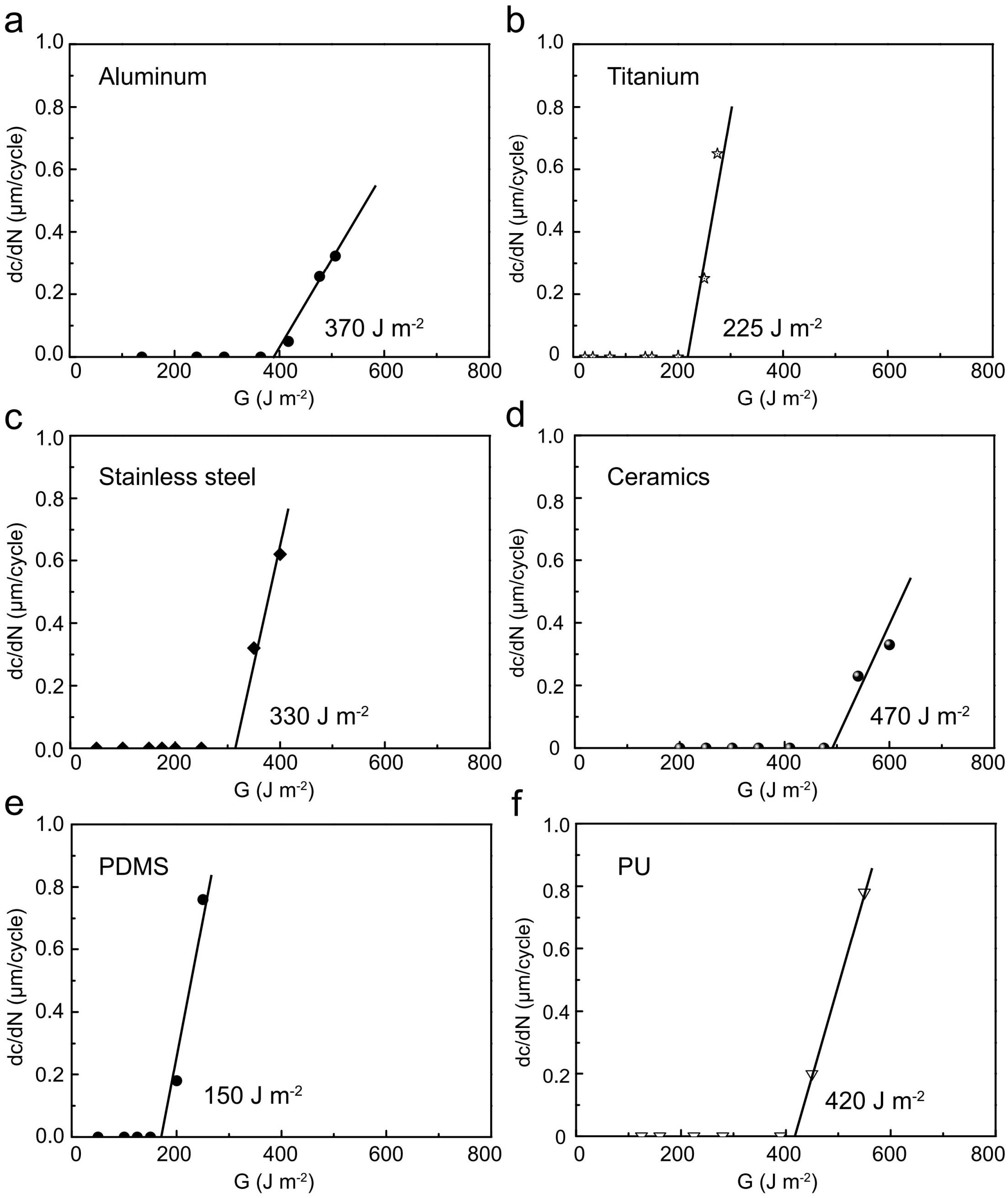

Supplementary Figure 12. Measurement of interfacial fatigue threshold of fatigue-resistant hydrogel adhesion (i.e., PVA) to various substrates. a. 370 J m^{-2} for aluminium, **b.** 225 J m^{-2} for titanium, **c.** 330 J m^{-2} for stainless steel, **d.** 470 J m^{-2} for ceramics, **e.** 150 J m^{-2} for PDMS, and **f.** 420 J m^{-2} for PU.

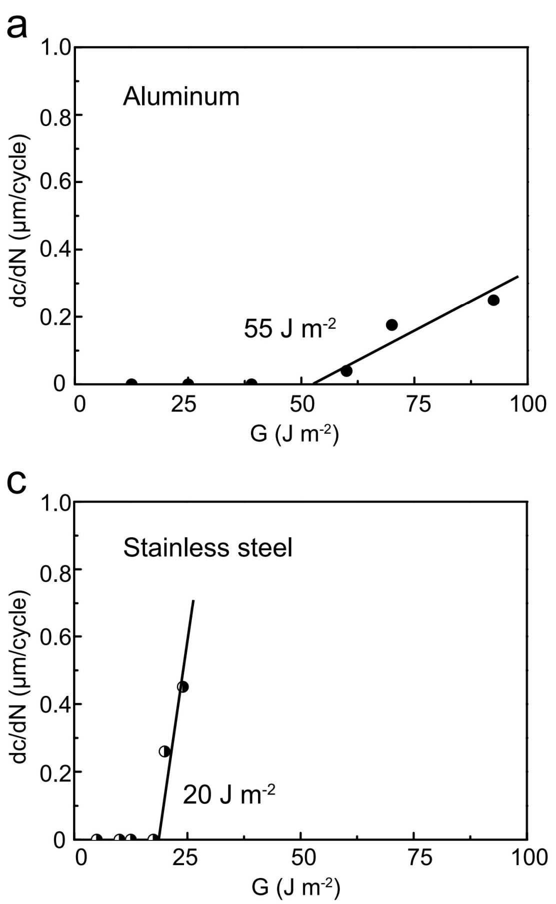

Supplementary Figure 13. Measurement of interfacial fatigue threshold of tough hydrogel adhesion (i.e., PAAm-alginate) to various substrates. a. 55 J m^{-2} for aluminium, **b.** 42 J m^{-2} for titanium, **c.** 20 J m^{-2} for stainless steel, and **d.** 58 J m^{-2} for ceramics.

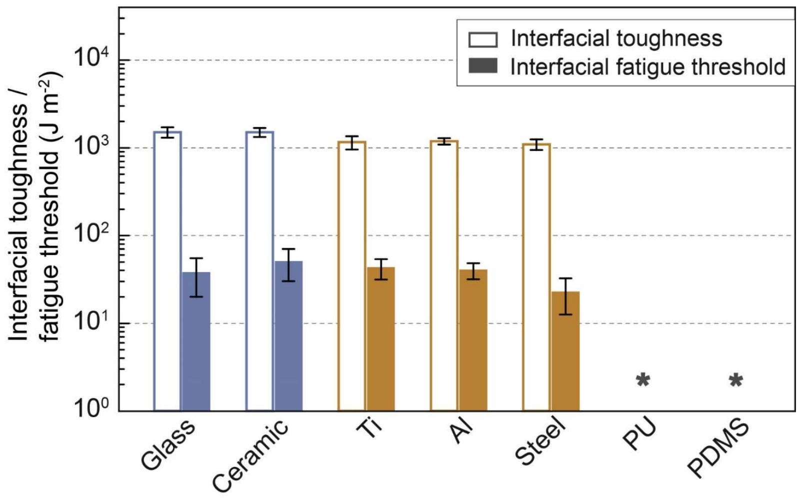

Supplementary Figure 14. Mechanical characterization of tough hydrogel adhesion. Summary of measured interfacial toughness and interfacial fatigue threshold of tough hydrogel adhesion on various solid substrates, including glass, ceramics, titanium (Ti), aluminium (Al), stainless steel, polydimethylsiloxane (PDMS) and polyurethane (PU). *Note: data not available since the tough hydrogel adhesion on PDMS and PU can not be achieved following the same protocol described[9]. Error bars = standard deviation (n=3).

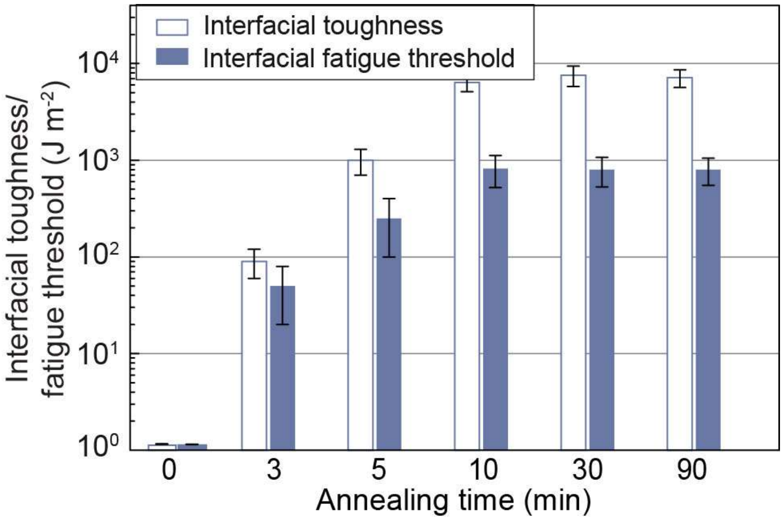

Supplementary Figure 15. Effect of annealing time on the fatigue-resistant hydrogel adhesion. Summary of interfacial toughness and interfacial fatigue threshold of fatigue-resistant hydrogel adhesion to glass with different annealing time. Error bars = standard deviation (n=3).

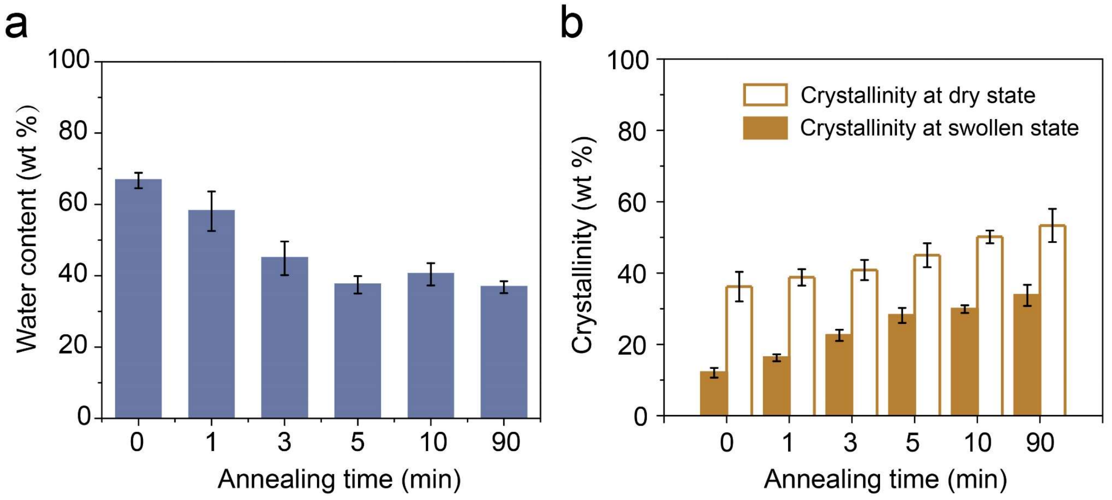

Supplementary Figure 16. Water content and crystallinity of fatigue-resistant hydrogel adhesion to glass with different annealing time. a. Summary of the water content of the PVA hydrogel adhesion on glass with annealing time of 0, 1, 3, 5, 10, 90 min. **b.** Summary of the crystallinity of the PVA hydrogel adhesion on glass with annealing time of 0, 1, 3, 5, 10, 90 min. Error bars = standard deviation (n=3).

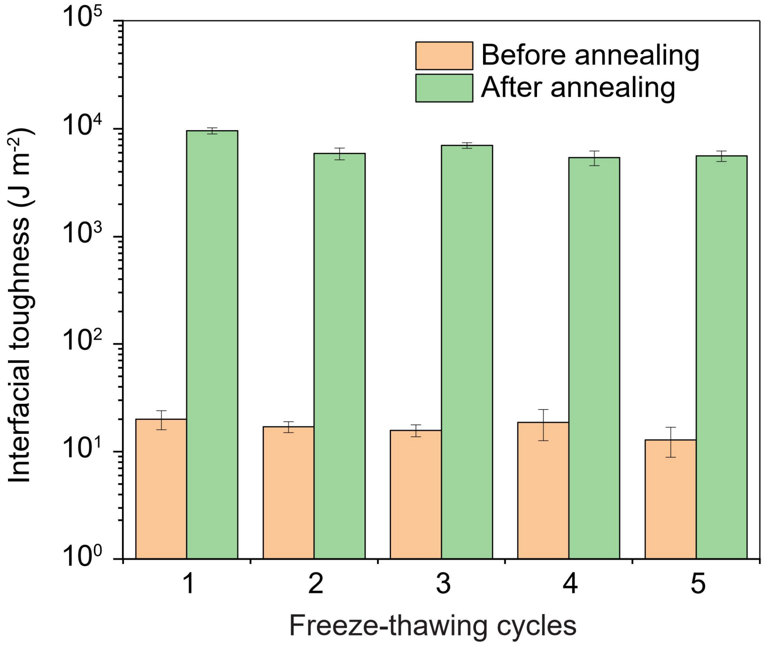

Supplementary Figure 17. Effect of freeze-thawing cycle number on the fatigue-resistant hydrogel adhesion. Summary of interfacial toughnesses of fatigue-resistant hydrogel adhesion to glass with different freeze-thawing cycles before and after 90-min annealing. Error bars = standard deviation (n=3).

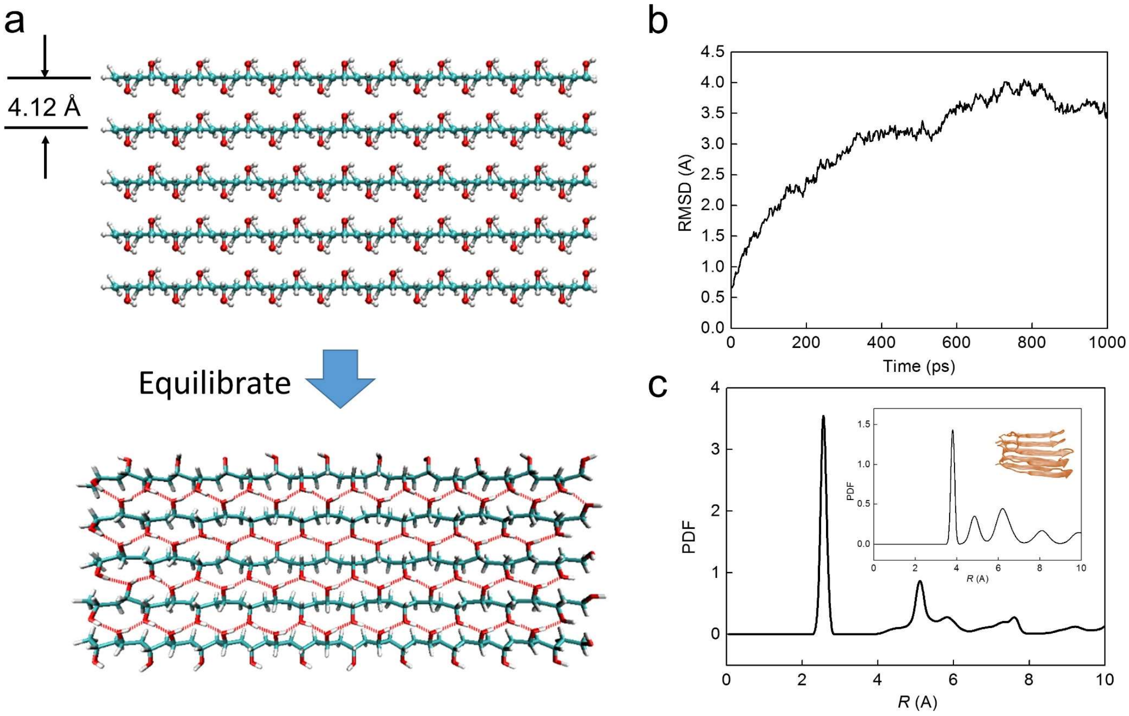

Supplementary Figure 18. **Modeling and equilibration of PVA nanocrystalline structure.** **a,** The initial fully atomic model of PVA nanocrystal and the fully equilibrate one. Different elements are colored with cyan for carbon, red for oxygen, white for hydrogen, and hydrogen bonds are highlighted by red dash lines. **b,** The root-mean-square deviation (RMSD) of all the atoms' coordinate deviated from its initial conformation during the first nanosecond of equilibration simulation, suggesting that the structure does not change too much from its initial conformation and starts to converge to a stable state. **c,** The atomic pair distribution function (PDF) of the carbon atoms within the equilibrated PVA nanocrystal, and the existence of the few peaks after the first peak (for the nearest C-C neighbor) suggests that the crystal structure is in analogy to the PDF of a beta-sheet structure for an amyloid protein as inserted.

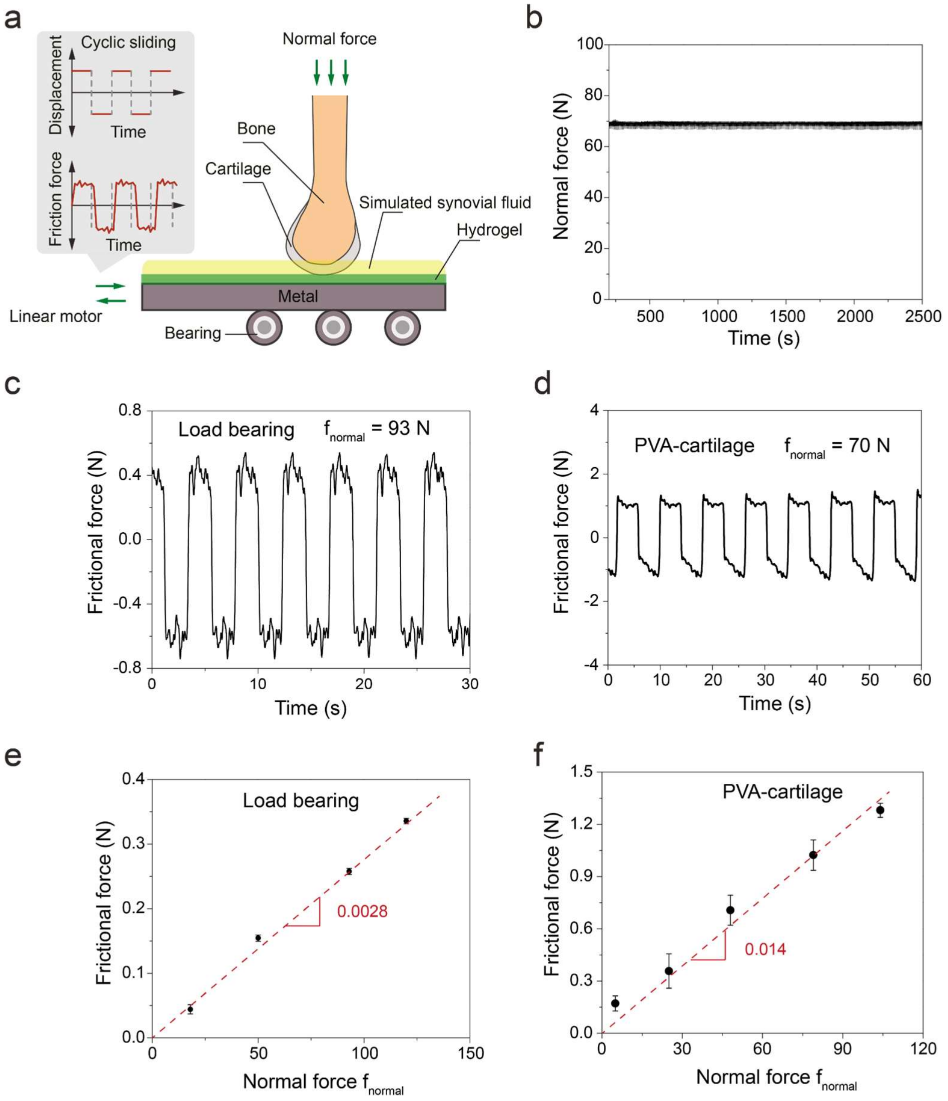

Supplementary Figure 19. Measurement of the friction coefficient (COF) of interfaces between cartilage and various substrates. a, Schematic illustration of set-up for measuring friction coefficient. **b,** Applied compressive normal force versus time. **c,** Frictional force versus time during the cyclic sliding at the interface between the metallic supporting plate and bearings at a set fore of 93 N. **d,** Frictional force versus time during cyclic sliding at the interface between cartilage and fatigue-resistant PVA hydrogel at a set force of 70 N. **e,** Steady-state frictional force versus applied normal force for the interface between metallic supporting plate and bearings. The slope gives the frictional coefficient of the interface between the metal substrate and bearings as 0.0028. **f,** Corrected frictional force versus applied normal force for the interface between PVA hydrogel and cartilage. The slope gives the frictional coefficient of the interface between the metal substrate and bearings as 0.014. Error bars = standard deviation (n=3).

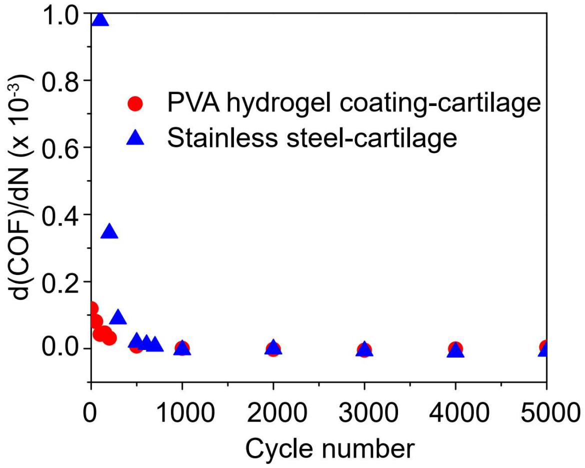

Supplementary Figure 20. The incremental frictional coefficient per cycle (d(COF)/dN) over cycles for PVA hydrogel coating against cartilage and bare stainless steel against cartilage at compressive force of 100 N.

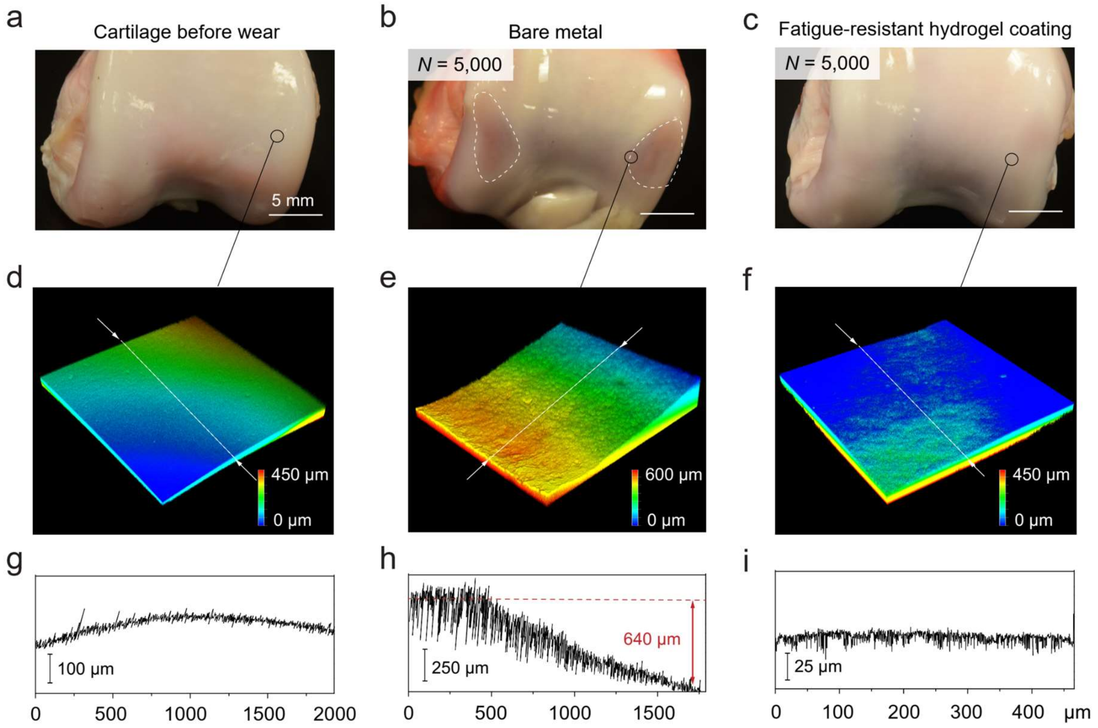

Supplementary Figure 21. **Wear of cartilages against different materials during reciprocating sliding. a-c**, Images of cartilage surfaces before (**a**) and after 5,000 cycles of reciprocating sliding against bare stainless steel (**b**), and fatigue-resistant hydrogel coating on stainless steel (**c**) with a normal compression force of 100 N (compressive stress of ~1 MPa). The regions circled in dotted white lines show the wear of cartilage surfaces against bare stainless steel (**b**). **d-f**, Topographic images obtained from confocal laser scanning microscopy for the contact zone of chicken cartilage before (**d**) and after 5,000 cycles of reciprocating sliding against bare stainless steel (**e**), and fatigue-resistant hydrogel coating (**f**). The colors in **d-f** indicate different height. Dot lines in **d-f** show the location of height profiles in **g-i**. **g-i**, Local height profiles of cartilage surfaces before (**g**) and after 5,000 cycles of reciprocating sliding against bare stainless steel (**h**), and fatigue-resistant hydrogel coating (**i**). Scale bars: 5 mm in **a-c**.

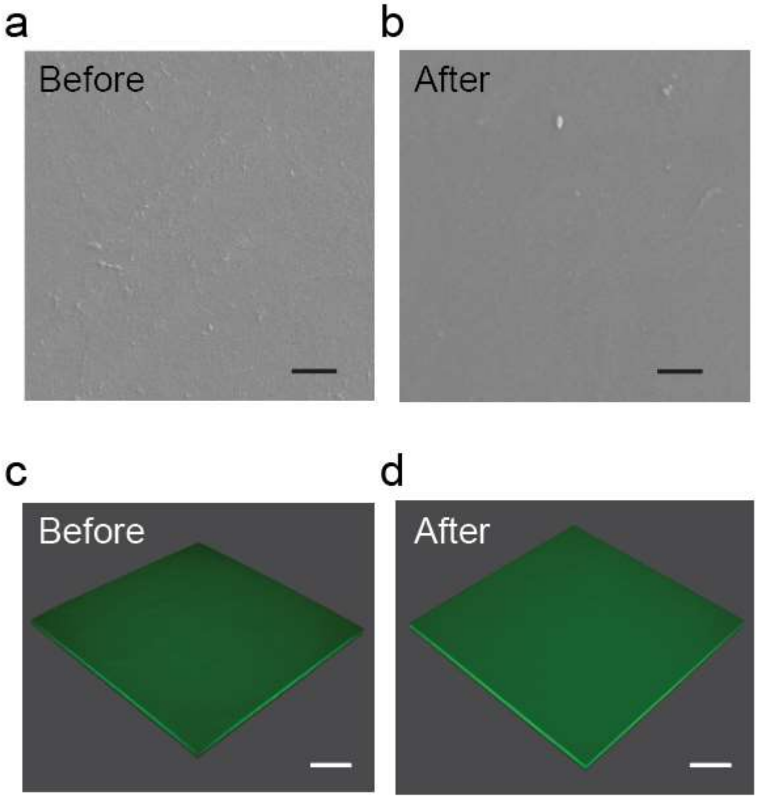

Supplementary Figure 22. Wear of fatigue-resistant hydrogel coating against cartilages during reciprocating sliding. a-b, Scanning electron microscopy images of fatigue-resistant hydrogel coating before (**a**) and after (**b**) 5,000 cycles of reciprocating sliding against cartilage. **c-d**, Confocal laser scanning microscopy images of fatigue-resistant hydrogel coating before (**c**) and after (**d**) 5,000 cycles of reciprocating sliding against cartilage. Scale bars: 20 μm in **a,b** and 500 μm in **c,d**.

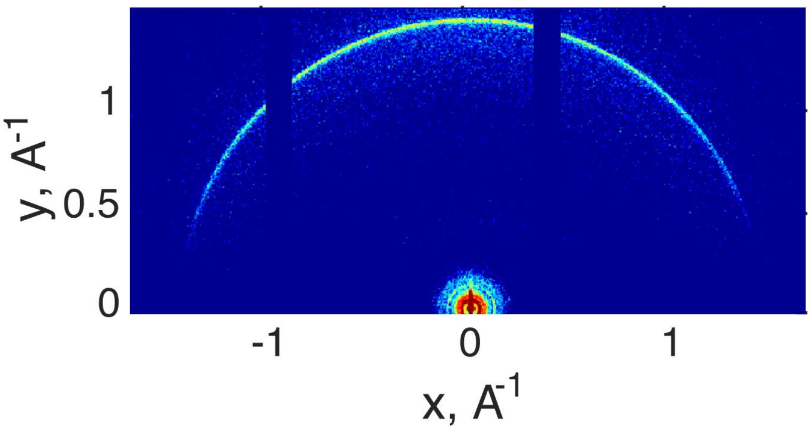

Supplementary Figure 23. GIWAXS pattern of a controlled sample PVA hydrogel on a polytetrafluoroethylene (PTFE) substrate. A uniform scattering ring is observed for PVA hydrogel on a PTFE substrate as a control, suggesting a randomly distributed crystalline domains, due to the absence of interfacial hydrogen bonding.

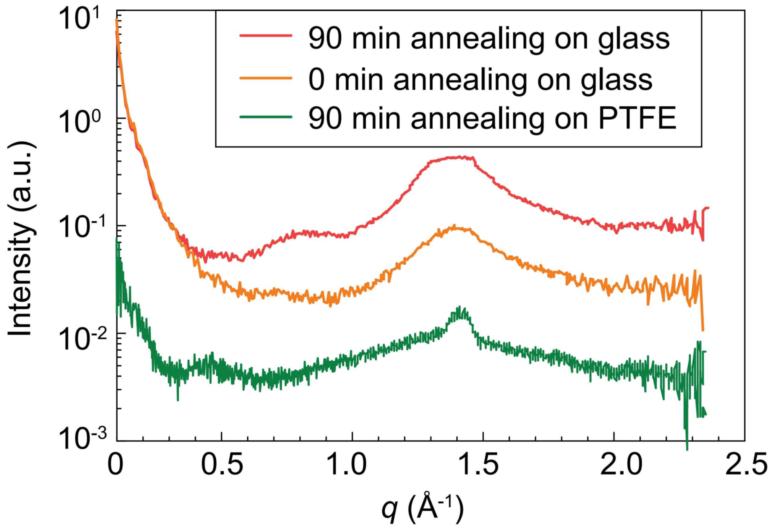

Supplementary Figure 24. GIWAXS curves for the PVA hydrogel adhesion to different substrates. GIWAXS curves for the PVA hydrogels before and after annealing on glass (100 °C, 90 min), and a PVA hydrogel annealed on PTFE (no hydrogen bonds at the interface between hydrogel and PTFE).

References

1. Peppas, N. A., Merrill, E. W. Differential scanning calorimetry of crystallized PVA hydrogels. *J. Appl. Polym. Sci.* **20**, 1457-1465 (1976).

2. Lin, S.*, et al.* Anti-fatigue-fracture hydrogels. *Sci. Adv.* **5**, eaau8528 (2019).

3. Yuk, H., Zhang, T., Lin, S., Parada, G. A., Zhao, X. Tough bonding of hydrogels to diverse non-porous surfaces. *Nat. Mater.* **15**, 190-196 (2016).
