# Supplementary material for: Fatigue-resistant adhesion of hydrogels
Source: Nat Commun. 2020 Feb 26;11:1071. doi: 10.1038/s41467-020-14871-3 (PMC7044439; doi:10.1038/s41467-020-14871-3)
Supplement: Supplementary file 2 — Description of Additional Supplementary Files [file 41467_2020_14871_MOESM2_ESM.docx]

**Description of Additional Supplementary Files**

**Title:** Supplementary Movie 1. **Description:** 90-degree peeling of fatigue-resistant hydrogel adhesion on a glass substrate.

**Title:** Supplementary Movie 2. **Description:** 90-degree peeling of fatigue-resistant hydrogel adhesion on a glass substrate under cyclic loading.

**Title:** Supplementary Movie 3. **Description:** Molecular dynamics simulation for pulling out a single PVA polymer chain from a nanocrystalline domain.

**Title:** Supplementary Movie 4. **Description:** Tough hydrogel coating on a stainless steel plate against cartilage, showing cohesive fracture at the 90th cycle of reciprocating sliding.

**Title:** Supplementary Movie 5. **Description:** Fatigue-resistant hydrogel coating on a stainless steel plate against cartilage, showing robust adhesion after 5,000 cycles of reciprocating sliding.
